# Synthesis, bioactivity, and molecular docking of novel arylpiperazine derivatives as potential AR antagonists

**DOI:** 10.3389/fchem.2022.947065

**Published:** 2022-08-15

**Authors:** Yueheng Qi, Hong Chen, Shijin Chen, Jianliang Shen, Jingguo Li

**Affiliations:** ^1^ Henan Provincial People’s Hospital, People’s Hospital of Zhengzhou University, Zhengzhou, Henan, China; ^2^ Luoyang Key Laboratory of Organic Functional Molecules, College of Food and Drug, Luoyang Normal University, Luoyang, Henan, China; ^3^ School of Ophthalmology & Optometry, School of Biomedical Engineering, Wenzhou Medical University, Wenzhou, Zhejiang, China

**Keywords:** prostate cancer, synthesis, antagonistic activity, binding affinities, molecular docking

## Abstract

Prostate cancer is one of the malignant tumors and the second most common malignant tumor in men. Clinically used androgen receptor (AR)–targeted drugs can antagonize androgen and inhibit tumor growth, but these drugs can cause serious resistance problems. To develop novel AR antagonists, 22 kinds of arylpiperazine derivatives were designed and synthesized, and the derivatives **5**, **8**, **12**, **19**, **21**, **22**, **25**, and **26** not only showed strong antagonistic potency (>55% inhibition) and binding affinities (IC_50_ <3 μM) to AR, but also showed stronger inhibitory activity to LNCaP cells *versus* PC-3 cells. Among them, derivative **21** exhibited the highest binding affinity for AR (IC_50_ = 0.65 μM) and the highest antagonistic potency (76.2% inhibition). Docking studies suggested that the derivative **21** is primarily bound to the AR-LBP site by the hydrophobic interactions. Overall, those results provided experimental methods for developing novel arylpiperazine derivatives as potent AR antagonists.

## Highlights

1. A series of arylpiperazine derivatives were synthesized
2. Antiproliferative (LNCaP cells *versus* PC-3 cells), AR antagonist activity, and AR binding affinity of arylpiperazine derivatives were investigated.
3. Some derivatives exhibited strong cytotoxic activities against LNCaP cells *versus* PC-3 cells and exhibited potent antagonistic potency against AR and AR binding affinities.
4. Molecular docking and SAR of arylpiperazine derivatives were also studied.

## Introduction

Prostate cancer (PCa) is one of the malignant tumors and the second most common malignant tumor in men that seriously endanger human health, with nearly 140,0000 new cases and 375,304 deaths in the year 2020 (Gandaglia et al., 2021; Sung et al., 2021). Androgen receptors (AR) are steroid receptors in the nuclear receptor superfamily and are highly expressed in prostate cancer cells, which play an important role in prostatic hyperplasia and growth. AR is involved in the progression of PCa, and AR is expressed to a certain extent in each stage of PCa (Bentel and Cardi, 1996; Bosland, 2000; Culig et al., 2002; Gelmann, 2002; Taplin and Balk, 2004; Dehm and Tindall, 2007). AR overexpression is also found in most castration-resistant prostate cancer (CRPC). The AR pathway still plays a key role in the growth and reproduction of CRPC, and the reactivation of the AR signaling pathway is a key driving force for the progression of CRPC. Therefore, AR has become an important target for the treatment of PCa.

**FIGURE 1:**
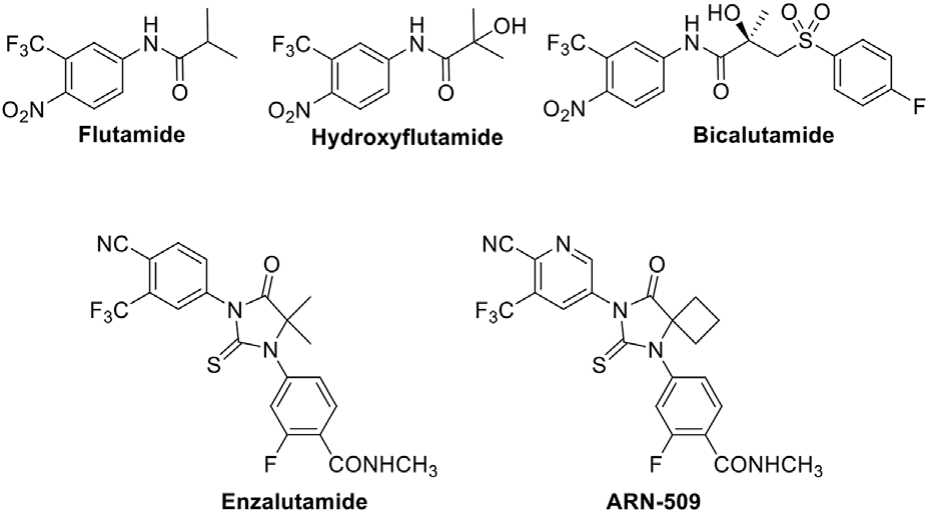
Structures of flutamide, hydroxyflutamide, bicalutamide, enzalutamide, and ARN-509.

At present, the main treatment methods for CRPC include endocrine therapy, chemotherapy, molecular targeted therapy and immunotherapy, and androgen-deprivation therapy is the standard treatment for advanced prostate cancer, but most patients progress to an incurable CRPC stage within 2–3 years. CRPC patients have a poor prognosis, difficult treatment, and are prone to metastasis, with median overall survival <2 years. Once PCa has metastasized, no obvious therapies exist (Zou et al., 2012; Dorff and Glode, 2013; Han et al., 2013; Loblaw et al., 2013). Although chemotherapy is most commonly used to treat advanced diseases (Beedassy and Cardi, 1999), and to inhibit tumor growth and prolong the life of patients, none of the conventional cancer therapy approaches have been shown to be effective against PCa. Thus, it is urgent to find and develop new therapeutic agents with obvious curative effects for the treatment of PCa. AR antagonists inhibit the activity of AR by directly binding and blocking the ligand-binding domain of AR and preventing androgens to exert the corresponding biological activity, thereby inhibiting the development of PCa. Although clinically used AR-targeted antagonists such as flutamide, hydroxyflutamide, bicalutamide, enzalutamide, and ARN-509 (Figure 1) can antagonize the function of androgens at the receptor level to inhibit tumor growth, these drugs produce serious adverse reactions and drug resistance after several years of targeted therapy. Therefore, as the second most frequent malignancy in men worldwide, it is an urgent problem to find and develop effective anti-drug AR-targeted antagonists for treating PCa.

**Scheme 1:**
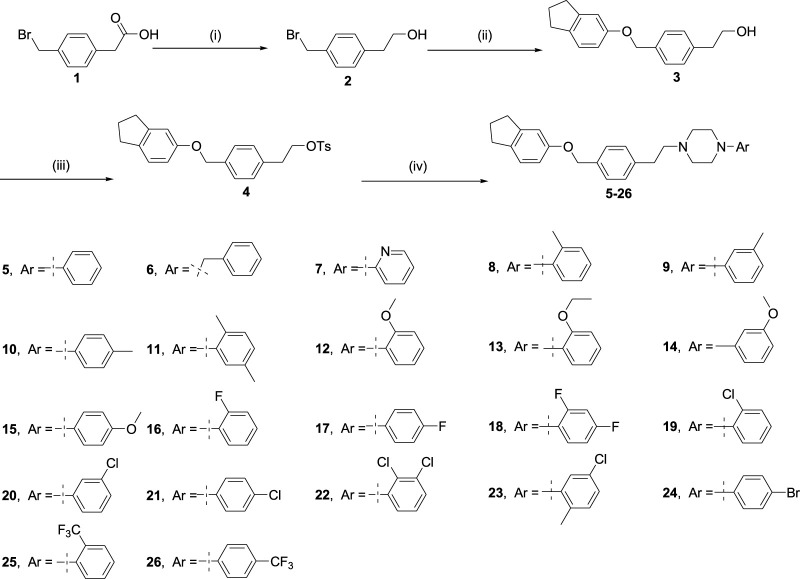
Reagents and conditions are as follows: (i) BH_3_.S(CH_3_)_2_, tetrahydrofuran, 11 h; (ii) 2,3-dihydro-1H-inden-5-ol, potassium carbonate, acetonitrile, 85^o^C, 16 h; (iii) TsCl, Et_3_N and 4-dimethylaminopyridine, methylene chloride, 0^o^C, 16 h; (iv) arylpiperazines, potassium carbonate, acetonitrile, 85^o^C, 16 h.

Piperazine is a six-membered heterocyclic compound, which is one of the most popular heterocyclic compounds for new drug candidates under development and existing marketed drugs (Chaudhary et al., 2006). Moreover, piperazine compounds have a broad spectrum of pharmacological activities because of their receptor-blocking (Leopoldo et al., 2007; Romeiro et al., 2011; Chen et al., 2012; Ananthan et al., 2014; Baran et al., 2014) and antiproliferative properties (Berardi et al., 2008; Lee et al., 2010; Abate et al., 2011; Cao et al., 2013; Lin et al., 2013; Liu et al., 2013; Arnatt et al., 2014; Guo et al., 2015). Arylpiperazine derivatives also have obvious AR antagonism with an IC_50_ of 0.11 μM, whereas the IC_50_ of bicaluramide is 50 μM. Results of animal experiments have shown that the mass of the prostate in rats is significantly reduced, and the concentration of serum testosterone is not significantly changed (Kinoyama et al., 2004; Kinoyama et al., 2005; Gupta et al., 2016). Recently, our group has also reported that some piperazine derivatives exhibit excellent inhibitory activity to AR (Chen et al., 2019a; Chen et al., 2019b). Inspired by these findings, in order to obtain more effective AR antagonists to treat PCa, here, a class of arylpiperazine derivatives (Scheme 1) was synthesized and their biological activity was evaluated. The structure–activity relationship (SAR) was also studied to further design the potent AR antagonists and develop the novel arylpiperazine derivatives. Compared to the reported derivatives, some arylpiperazine derivatives exhibited relatively excellent bioactivity.

## Materials and methods

### Materials and instruments

Anhydrous tetrahydrofuran was dried using standard experimental procedures. Chemical reagents and other solvents were purchased from Energy Chemical. Melting points of compounds were obtained using an SGW X-4 micromelting point instrument (Uncorrected). NMR spectra were recorded on a Bruker AVANCE-500 MHz instrument in CDCl_3_. The chemical shifts are given in ppm, and *J*-values are reported in Hz. High-resolution mass spectra (AB Sciex X500R QTOF) of the intermediates and the final compounds were obtained equipped with an ESI source.

### Synthesis of 2-(4-(bromomethyl)phenyl)ethanol (2)

The intermediate **2** was obtained using previous literature methods (Chen et al., 2018).

### 2-(4-((2,3-dihydro-1H-inden-5-yloxy)methyl)phenyl)ethanol (3)

The intermediate **3** was obtained using methods outlined in previous literature (Chen et al., 2018). White solid (ethyl acetate). Yield: 60% (from compound **1)**; Mp, 45.8–46.5 ^o^C; ^1^H NMR (500 MHz, CDCl_3_) δ 7.42 (d, *J* = 7.9 Hz, 2H), 7.28 (d, *J* = 7.9 Hz, 2H), 7.16 (d, *J* = 8.0 Hz, 1H), 6.91 (d, *J* = 2.0 Hz, 1H), 6.80 (dd, *J* = 8.0, 2.0 Hz, 1H), 5.05 (s, 2H), 3.89 (t, *J* = 6.6 Hz, 2H), 2.93–2.87 (m, 6H), 2.08–2.14 (m, 2H); HRMS (ESI) m/z [M+1]^+^: calcd for C_18_H_20_O_2,_ 269.1536, found, 269.1536.

### 4-((2,3-dihydro-1H-inden-5-yloxy)methyl)phenethyl 4-methylbenzenesulfonate (4)

The intermediate **4** was obtained using previously reported methods (Chen et al., 2018). White solid (ethyl acetate). Yield: 95%. Mp 63.2–64.3 ^o^C; ^1^H NMR (500 MHz, CDCl_3_) δ 7.70 (d, *J* = 8.2 Hz, 2H), 7.34 (d, *J* = 8.0 Hz, 2H), 7.30 (d, *J* = 8.4 Hz, 2H), 7.14 (d, *J* = 8.0 Hz, 3H), 6.89 (d, *J* = 2.0 Hz, 1H), 6.78 (dd, *J* = 8.2, 2.0 Hz, 1H), 5.02 (s, 2H), 4.23 (t, *J* = 7.0 Hz, 2H), 2.99 (t, *J* = 7.0 Hz, 2H), 2.89–2.83 (m, 4H), 2.45 (s, 3H), 2.07–2.13 (m, 2H); HRMS (ESI) m/z [M+1]^+^: calcd for C_25_H_26_O_4_S, 423.1625, found, 423.1621.

### General procedure for the synthesis of arylpiperazine derivative 5–26

To a solution of **4** (100 mg, 0.23 mmol) in acetonitrile (10 ml) was added arylpiperazine (1.2 equiv) and potassium carbonate (6.0 equiv), and the reaction mixture was stirred at 85 ^o^C for 16 h. The inorganic solids were filtered by the Buchner funnel, and the remaining filtrate was evaporated *in vacuo*. Obtained crude products were purified using column chromatography (V_PE_: V_EA_ = 15:1) to give the respective product (**5**–**26**).

#### 1-(4-((2,3-dihydro-1H-inden-5-yloxy)methyl)phenethyl)-4-phenylpiperazine (**5**)

White solid (ethyl acetate); Yield: 64%; Mp 82.2–83.5 ^o^C; ^1^H NMR (500 MHz, CDCl_3_) δ 7.42 (d, *J* = 7.9 Hz, 2H), 7.36–7.28 (m, 4H), 7.17 (d, *J* = 8.2 Hz, 1H), 7.00 (d, *J* = 8.2 Hz, 2H), 6.92 (t, *J* = 7.2 Hz, 2H), 6.82 (dd, *J* = 8.2, 2.2 Hz, 1H), 5.06 (s, 2H), 3.29 (t, *J* = 5.0 Hz, 4H), 2.95–2.85 (m, 6H), 2.80–2.67 (m, 6H), 2.12 (m, 2H).^13^C NMR (126 MHz, CDCl_3_) δ 157.85, 151.35, 145.76, 139.95, 136.47, 135.23, 129.16, 128.95, 127.74, 124.79, 119.77, 116.11, 112.86, 110.96, 70.12, 60.48, 53.29, 49.20, 33.38, 33.23, 32.04, 25.89; HRMS (ESI) m/z [M+1]^+^: calcd for C_28_H_32_N_2_O, 413.2587, found, 413.2584.

#### 1-(4-((2,3-dihydro-1H-inden-5-yloxy)methyl)phenethyl)-4-benzylpiperazine (**6**)

White solid (ethyl acetate); Yield: 60%; Mp 71.1–73.3 ^o^C; ^1^H NMR (500 MHz, CDCl_3_) δ 7.39–7.28 (m, 7H), 7.21 (d, *J* = 8.0 Hz, 2H), 7.11 (d, *J* = 8.1 Hz, 1H), 6.86 (d, *J* = 2.0 Hz, 1H), 6.75 (dd, *J* = 8.2, 2.0 Hz, 1H), 4.99 (s, 2H), 3.53 (s, 2H), 2.88–2.809 (m, 6H), 2.77–2.31 (m, 10H), 2.11–2.01 (m, 2H);^13^C NMR (126 MHz, CDCl_3_) δ 157.93, 145.85, 140.08, 138.11, 136.56, 135.27, 129.39, 129.01, 128.36, 127.80, 127.21, 124.86, 112.94, 111.05, 70.22, 63.17, 60.54, 53.25, 53.09, 33.38, 33.31, 32.12, 25.97; HRMS (ESI) m/z [M+1]^+^: calcd for C_29_H_34_N_2_O, 427.2744, found, 427.2742.

#### 1-(4-((2,3-dihydro-1H-inden-5-yloxy)methyl)phenethyl)-4-(pyridin-2-yl)piperazine (**7**)

White solid (ethyl acetate); Yield: 90%; Mp 90.1–91.8 ^o^C; ^1^H NMR (500 MHz, CDCl_3_) δ 8.24 (dd, *J* = 5.0, 1.5Hz, 1H), 7.56–7.47 (m, 1H), 7.39 (d, *J* = 7.9 Hz, 2H), 7.28 (d, *J* = 7.9 Hz, 2H), 7.15 (d, *J* = 8.2 Hz, 1H), 6.90 (br s, 1H), 6.79 (dd, *J* = 8.2, 2.0 Hz, 1H), 6.74–6.60 (m, 2H), 5.03 (s, 2H), 3.62 (t, *J* = 5.0 Hz, 4H), 2.95–2.81 (m, 6H), 2.71–2.68 (m, 6H), 2.16–2.03 (m, 2H);^13^C NMR (126 MHz, CDCl_3_) δ 159.55, 157.81, 148.00, 145.75, 139.85, 137.50, 136.45, 135.22, 128.94, 127.73, 124.77, 113.39, 112.82, 110.93, 107.13, 70.09, 60.47, 53.01, 45.19, 33.24, 33.21, 32.02, 29.74, 25.87, 22.73; HRMS (ESI) m/z [M+1]^+^: calcd for C_27_H_31_N_3_O, 414.2540, found, 414.2536.

#### 1-(4-((2,3-dihydro-1H-inden-5-yloxy)methyl)phenethyl)-4-o-tolylpiperazine (**8**)

White solid (ethyl acetate); Yield: 85%; Mp 62.3–63.1 ^o^C; ^1^H NMR (500 MHz, CDCl_3_) δ 7.37 (d, *J* = 8.0 Hz, 2H), 7.25 (d, *J* = 8.0 Hz, 2H), 7.18 (*J* = 7.6 Hz, 2H), 7.12 (d, *J* = 8.2 Hz, 1H), 7.05 (d, *J* = 7.5 Hz, 1H), 6.99 (td, *J* = 7.5, 2.0 Hz, 1H), 6.87 (d, *J* = 2.0 Hz, 1H), 6.77 (dd, *J* = 8.2, 2.0 Hz, 1H), 5.01 (s, 2H), 3.00 (t, *J* = 5.0 Hz, 4H), 2.94–2.78 (m, 6H), 2.71–2.67 (m, 6H), 2.32 (s, 3H), 2.15–1.97 (m, 2H); ^13^C NMR (126 MHz, CDCl_3_) δ 158.06, 151.68, 146.01, 140.19, 136.71, 135.45, 132.87, 131.33, 129.20, 128.00, 126.87, 125.02, 123.45, 119.29, 113.07, 111.17, 70.35, 60.83, 53.98, 51.89, 33.58, 33.46, 32.27, 26.12, 18.17; HRMS (ESI) m/z [M+1]^+^: calcd for C_29_H_34_N_2_O, 427.2744, found, 427.2742.

#### 1-(4-((2,3-dihydro-1H-inden-5-yloxy)methyl)phenethyl)-4-m-tolylpiperazine (**9**)

White solid (ethyl acetate); Yield: 65%; Mp 61.2–62.7 ^o^C; ^1^H NMR (500 MHz, CDCl_3_) δ 7.35 (d, *J* = 8.0 Hz, 2H), 7.23 (d, *J* = 8.0 Hz, 2H), 7.15 (t, *J* = 7.8 Hz, 1H), 7.10 (d, *J* = 8.0 Hz, 1H), 6.86 (d, *J* = 2.0 Hz, 1H), 6.76–6.74 (m, 3H), 6.69 (d, *J* = 7.6 Hz, 1H), 4.99 (s, 2H), 3.22 (t, *J* = 5.0 Hz, 4H), 2.88–2.81 (m, 6H), 2.70–2.63 (m, 6H), 2.32 (s, 3H), 2.09–2.03 (m, 2H);^13^C NMR (126 MHz, CDCl_3_) δ 157.88, 151.42, 145.81, 139.94, 138.88, 136.51, 135.28, 129.06, 129.00, 127.80, 124.84, 120.77, 117.03, 113.31, 112.89, 110.99, 70.14, 60.50, 53.31, 49.26, 33.34, 33.28, 32.09, 29.81, 25.94, 21.90; HRMS (ESI) m/z [M+1]^+^: calcd for C_29_H_34_N_2_O, 427.2744, found, 427.2740.

#### 1-(4-((2,3-dihydro-1H-inden-5-yloxy)methyl)phenethyl)-4-p-tolylpiperazine (**10**)

White solid (ethyl acetate); Yield: 68%; Mp 105.1–106.3 ^o^C; ^1^H NMR (500 MHz, CDCl_3_) δ 7.40 (d, *J* = 8.0 Hz, 2H), 7.28 (d, *J* = 8.0 Hz, 2H), 7.15 (d, *J* = 8.2 Hz, 1H), 7.12 (d, *J* = 8.2 Hz, 2H), 6.91–6.89 (m, 3H), 6.80 (dd, *J* = 8.2, 2.0 Hz, 1H), 5.04 (s, 2H), 3.24 (t, *J* = 5.0 Hz, 4H), 2.95–2.84 (m, 6H), 2.81–2.68 (m, 6H), 2.31 (s, 3H), 2.15–2.06 (m, 2H);^13^C NMR (126 MHz, CDCl_3_) δ 157.80, 149.18, 145.75, 139.80, 136.46, 135.24, 129.68, 129.38, 128.94, 127.74, 124.76, 116.50, 112.82, 110.93, 70.09, 60.42, 53.24, 49.67, 33.21, 32.01.25.86.20.46; HRMS (ESI) m/z [M+1]^+^: calcd for C_29_H_34_N_2_O, 427.2744, 427.2741.

#### 1-(4-((2,3-dihydro-1H-inden-5-yloxy)methyl)phenethyl)-4-(2,5-dimethylphenyl)piperazine (**11**)

White solid (ethyl acetate); Yield: 46%; Mp 70.4–71.5 ^o^C; ^1^H NMR (500 MHz, CDCl_3_) δ 7.41 (d, *J* = 8.0 Hz, 2H), 7.29 (d, *J* = 8.0 Hz, 2H), 7.16 (d, *J* = 8.2 Hz, 1H), 7.11 (d, *J* = 7.8 Hz, 1H), 6.92 (d, *J* = 2.0 Hz, 1H), 6.90 (br s, 1H), 6.85 (d, *J* = 7.6 Hz, 1H), 6.81 (dd, *J* = 8.2, 2.0 Hz, 1H), 5.05 (s, 2H), 3.02 (t, *J* = 5.0 Hz, 4H), 2.94–2.87 (m, 6H), 2.75–2.71 (m, 6H), 2.36 (s, 3H), 2.31 (s, 3H), 2.15–2.08 (m, 2H);^13^C NMR (126 MHz, CDCl_3_) δ 157.85, 151.31, 145.75, 140.01, 136.46, 136.14, 135.20, 130.91, 129.30, 128.95, 127.73, 124.77, 123.80, 119.78, 112.86, 110.96, 70.13, 60.53, 53.72, 51.64, 33.29, 33.22, 32.03, 25.87, 21.24, 17.51; HRMS (ESI) m/z [M+1]^+^: calcd for C_30_H_36_N_2_O, 441.2900, found, 441.2896.

#### 1-(4-((2,3-dihydro-1H-inden-5-yloxy)methyl)phenethyl)-4-(2-methoxyphenyl)piperazine (**12**)

White solid (ethyl acetate); Yield: 50%; Mp 77.3–78.5; ^1^H NMR (500 MHz, CDCl_3_) δ 7.40 (d, *J* = 7.9 Hz, 2H), 7.28 (d, *J* = 7.9 Hz, 2H), 7.15 (d, *J* = 8.1 Hz, 1H), 7.07–6.95 (m, 3H), 6.93–6.88 (m, 2H), 6.80 (dd, *J* = 8.2, 2.0 Hz, 1H), 5.04 (s, 2H), 3.90 (s, 3H), 3.19 (br s, 4H), 2.93–2.86 (m, 6H), 2.81–2.72 (m, 6H), 2.13–2.07 (m, 2H);^13^C NMR (126 MHz, CDCl_3_) δ 157.83, 152.30, 145.73, 141.28, 139.91, 136.45, 135.21, 128.94, 127.72, 124.76, 123.00, 121.04, 118.27, 112.85, 111.22, 110.95, 70.11, 60.49, 55.39, 53.40, 50.56, 33.21, 32.02, 29.73, 25.86; HRMS(ESI) m/z [M+1]^+^: calcd for C_29_H_34_N_2_O_2_, 443.2693, found, 443.2691.

#### 1-(4-((2,3-dihydro-1H-inden-5-yloxy)methyl)phenethyl)-4-(2-ethoxyphenyl)piperazine (**13**)

White solid (ethyl acetate); Yield: 54%; Mp 65.2–66.1 ^o^C; ^1^H NMR (500 MHz, CDCl_3_) δ 7.40 (d, *J* = 8.0 Hz, 2H), 7.29 (d, *J* = 8.0 Hz, 2H), 7.15 (d, *J* = 8.2 Hz, 1H), 7.04–6.94 (m, 3H), 6.93–6.87 (m, 2H), 6.80 (dd, *J* = 8.2, 2.0 Hz, 1H), 5.04 (s, 2H), 4.11 (q, *J* = 7.0 Hz, 2H), 3.22 (br s, 4H), 2.96–2.84 (m, 6H), 2.85–2.69 (m, 6H), 2.16–2.05 (m, 2H), 1.51 (t, *J* = 7.0 Hz, 3H);^13^C NMR (126 MHz, CDCl_3_) δ 157.84, 151.60, 145.74, 141.33, 139.93, 136.45, 135.21, 128.95, 127.72, 124.76, 122.81, 121.05, 118.22, 112.85, 112.52, 110.96, 77.34, 77.08, 76.83, 70.12, 63.59, 60.50, 53.41, 50.46, 33.21, 32.02, 25.87, 14.99; HRMS (ESI) m/z [M+1]^+^: calcd for C_30_H_36_N_2_O_2_, 457.2850, found, 457.2845.

#### 1-(4-((2,3-dihydro-1H-inden-5-yloxy)methyl)phenethyl)-4-(3-methoxyphenyl)piperazine (**14**)

White solid (ethyl acetate); Yield: 68%; Mp 74.2–75.4 ^o^C; ^1^H NMR (500 MHz, CDCl_3_) δ 7.40 (d, *J* = 7.8 Hz,2H), 7.28 (d, *J* = 7.8 Hz, 2H), 7.21 (t, *J* = 8.2 Hz, 1H), 7.15 (d, *J* = 8.2 Hz, 1H), 6.90 (br s, 1H), 6.79 (dd, *J* = 8.2, 2.0 Hz, 1H), 6.59 (dd, *J* = 8.2, 2.0 Hz, 1H), 6.52 (br s, 1H), 6.46 (dd, *J* = 8.1, 2.0 Hz, 1H), 5.04 (s, 2H), 3.83 (s, 3H), 3.28 (t, *J* = 5.0 Hz, 4H)), 2.99–2.82 (m, 6H), 2.80–2.63 (m, 6H), 2.17–2.03 (m, 2H);^13^C NMR (126 MHz, CDCl_3_) δ 160.61, 157.81, 152.67, 145.74, 139.81, 136.46, 135.25, 129.81, 128.92, 127.73, 124.76, 112.83, 110.94, 108.91, 104.50, 102.57, 70.10, 60.39, 55.21, 53.16, 49.02, 33.24, 33.20, 32.01, 29.73, 25.86; HRMS (ESI) m/z [M+1]^+^: calcd for C_29_H_34_N_2_O_2_, 443.2693, found, 443.2689.

#### 1-(4-((2,3-dihydro-1H-inden-5-yloxy)methyl)phenethyl)-4-(4-methoxyphenyl)piperazine (**15**)

White solid (ethyl acetate); Yield: 45%; Mp 78.1–79.3 ^o^C; ^1^H NMR (500 MHz, CDCl_3_) δ 7.39 (d, *J* = 7.9 Hz, 2H), 7.28 (d, *J* = 7.9 Hz, 2H), 7.14 (d, *J* = 8.2 Hz, 1H), 6.95 (d, *J* = 9.0 Hz, 2H), 6.89 (br s, 1H), 6.87 (t, *J* = 7.9 Hz, 2H), 6.79 (dd, *J* = 8.1, 2.0 Hz, 1H), 5.03 (s, 2H), 3.80 (s, 3H), 3.23 (t, *J* = 5.0 Hz, 4H), 2.93–2.84 (m, 6H), 2.81–2.67 (m, 6H), 2.13–2.06 (m, 2H); ^13^C NMR (126 MHz, CDCl_3_) δ 157.82, 153.90, 145.74, 145.67, 139.82, 136.46, 135.24, 128.92, 127.72, 124.75, 118.28, 114.47, 112.84, 110.94, 70.10, 60.37, 55.59, 53.30, 50.58, 33.20, 32.01, 29.72, 25.85; HRMS (ESI) m/z [M+1]^+^: calcd for C_29_H_34_N_2_O_2_, 443.2693, found, 443.2690.

#### 1-(4-((2,3-dihydro-1H-inden-5-yloxy)methyl)phenethyl)-4-(2-fluorophenyl)piperazine (**16**)

White solid (ethyl acetate); Yield: 90%; Mp 64.2–65.3 ^o^C; ^1^H NMR (500 MHz, CDCl_3_) δ 7.41 (d, *J* = 8.0 Hz, 2H), 7.28 (d, *J* = 8.0 Hz, 2H), 7.15 (d, *J* = 8.1 Hz, 1H), 7.13–6.94 (m, 4H), 6.91 (d, *J* = 2.0 Hz, 1H), 6.80 (dd, *J* = 8.2, 2.0 Hz, 1H), 5.04 (s, 2H), 3.19 (t, *J* = 5.0 Hz, 4H), 2.96–2.84 (m, 6H), 2.83–2.66 (m, 6H), 2.14–2.08 (m, 2H);^13^C NMR (126 MHz, CDCl_3_) δ 157.82, 145.76, 139.95, 136.46, 135.19, 128.95, 127.74, 124.77, 124.51, 124.49, 118.96, 118.94, 116.22, 116.05, 112.83, 110.93, 70.09, 60.50, 53.31, 50.59, 50.56, 33.34, 33.22, 32.02, 25.88; HRMS (ESI) m/z [M+1]^+^: calcd for C_28_H_31_FN_2_O, 431.2493, found, 431.2492.

#### 1-(4-((2,3-dihydro-1H-inden-5-yloxy)methyl)phenethyl)-4-(4-fluorophenyl)piperazine (**17**)

White solid (ethyl acetate); Yield: 52%; Mp 79.8–80.5 ^o^C; ^1^H NMR (500 MHz, CDCl_3_) δ 7.40 (d, *J* = 7.9 Hz, 2H), 7.28 (d, *J* = 7.9 Hz, 2H), 7.15 (d, *J* = 8.2 Hz, 1H), 7.00 (t, *J* = 8.5 Hz, 2H), 6.94–6.92 (m, 2H), 6.90 (br s, 1H), 6.80 (dd, *J* = 8.2, 2.0 Hz, 1H), 5.04 (s, 2H), 3.20 (t, *J* = 5.0 Hz, 4H), 2.97–2.84 (m, 6H), 2.82–2.66 (m, 6H), 2.15–2.06 (m, 2H);^13^C NMR (126 MHz, CDCl_3_) δ 158.18, 157.82, 156.28, 147.97, 145.75, 139.83, 136.47, 135.25, 128.92, 127.73, 124.77, 117.90, 117.84, 115.63, 115.45, 112.84, 110.94, 70.10, 60.36, 53.23, 50.17, 33.29, 33.21, 32.02, 29.73, 25.86; HRMS (ESI) m/z [M+1]^+^: calcd for C_28_H_31_FN_2_O, 431.2493, found, 431.2487.

#### 1-(4-((2,3-dihydro-1H-inden-5-yloxy)methyl)phenethyl)-4-(2,4-difluorophenyl)piperazine (**18**)

White solid (ethyl acetate); Yield: 43%; Mp 86.1–86.5 ^o^C; ^1^H NMR (500 MHz, CDCl_3_) δ 7.40 (d, *J* = 8.0 Hz, 2H), 7.28 (d, *J* = 8.0 Hz, 2H), 7.15 (d, *J* = 8.2 Hz, 1H), 6.99–6.92 (m, 1H), 6.90 (d, *J* = 5.0 Hz, 1H), 6.88–6.77 (m, 3H), 5.04 (s, 2H), 3.12 (d, *J* = 5.0 Hz, 4H), 2.93–2.86 (m, 6H), 2.81–2.67 (m, 6H), 2.16–2.05 (m, 2H);^13^C NMR (126 MHz, CDCl_3_) δ 158.06, 145.99, 140.13, 136.70, 135.46, 129.16, 127.97, 125.00, 119.73, 119.69, 119.65, 119.62, 113.07, 111.17, 111.04, 111.01, 110.87, 110.84, 105.15, 104.95, 104.75, 70.33, 60.65, 53.50, 51.18, 51.16, 33.54, 33.45, 32.25, 26.10; HRMS (ESI) m/z [M+1]^+^: calcd for C_28_H_30_F_2_N_2_O, 449.2399, found, 449.2398.

#### 1-(4-((2,3-dihydro-1H-inden-5-yloxy)methyl)phenethyl)-4-(2-chlorophenyl)piperazine (**19**)

White solid (ethyl acetate); Yield: 43%; Mp 91.4–92.1 ^o^C; ^1^H NMR (500 MHz, CDCl_3_) δ 7.40 (d, *J* = 7.8 Hz, 3H), 7.28 (d, *J* = 7.8 Hz, 2H), 7.25 (t, *J* = 7.6 Hz, 1H),7.15 (d, *J* = 8.2 Hz, 1H), 7.10 (d, *J* = 7.6 Hz, 1H), 7.01 (t, *J* = 7.6 Hz, 1H), 6.90 (br s, 1H), 6.80 (dd, *J* = 8.2, 2.0 Hz, 1H), 5.04 (s, 2H), 3.16 (br s, 4H), 2.96–2.86 (m, 6H), 2.84–2.65 (m, 6H), 2.14–2.05 (m, 2H);^13^C NMR (126 MHz, CDCl_3_) δ 157.83, 149.30, 145.74, 139.97, 136.46, 135.20, 130.67, 128.93, 128.82, 127.72, 127.62, 124.76, 123.71, 120.41, 112.84, 110.95, 70.12, 60.46, 53.38, 51.22, 33.35, 33.21, 32.02, 25.86; HRMS (ESI) m/z [M+1]^+^: calcd for C_28_H_31_ClN_2_O, 447.2198, found, 447.2195.

#### 1-(4-((2,3-dihydro-1H-inden-5-yloxy)methyl)phenethyl)-4-(3-chlorophenyl)piperazine (**20**)

White solid (ethyl acetate); Yield: 71%; Mp 83.2–84.3 ^o^C; ^1^H NMR (500 MHz, CDCl_3_) δ 7.40 (d, *J* = 7.9 Hz, 2H), 7.28 (d, *J* = 2.3 Hz, 1H), 7.20 (t, *J* = 8.1 Hz, 1H), 7.15 (d, *J* = 8.2 Hz, 1H), 6.92 (t, *J* = 2.0 Hz, 1H), 6.90 (s, 1H), 6.86–6.79 (m, 3H), 5.04 (s, 2H), 3.35–3.18 (m, 4H), 2.99–2.81 (m, 6H), 2.71 (dd, *J* = 16.0, 7.0 Hz, 6H), 2.16–2.05 (m, 2H);^13^C NMR (126 MHz, CDCl_3_) δ 157.81, 152.29, 145.75, 139.72, 136.48, 135.29, 134.99, 130.05, 128.92, 127.74, 124.76, 119.36, 115.81, 113.91, 112.84, 110.94, 70.10, 60.30, 53.00, 48.62, 33.21, 32.02, 29.73, 25.86; HRMS (ESI) m/z [M+1]^+^: calcd for C_28_H_31_ClN_2_O, 447.2198, found, 447.2196.

#### 1-(4-((2,3-dihydro-1H-inden-5-yloxy)methyl)phenethyl)-4-(4-chlorophenyl)piperazine (**21**)

White solid (ethyl acetate); Yield: 57%; Mp 117.2–118.6 ^o^C; ^1^H NMR (500 MHz, CDCl_3_) δ 7.40 (d, *J* = 7.9 Hz, 2H), 7.29–7.23 (m, 4H), 7.14 (d, *J* = 8.2 Hz, 1H), 6.90 (br s, 1H), 6.88 (d, *J* = 8.2 Hz, 2H), 6.79 (dd, *J* = 8.2, 2.0 Hz, 1H), 5.04 (s, 2H), 3.23 (t, *J* = 5.0, 4H), 2.89 (m, 6H), 2.79–2.65 (m, 6H), 2.15–2.05 (m, 2H);^13^C NMR (126 MHz, CDCl_3_) δ 157.82, 149.93, 145.74, 139.82, 136.47, 135.26, 128.97, 128.91, 127.72, 124.76, 124.57, 117.25, 112.84, 110.94, 70.10, 60.33, 53.07, 49.16, 33.29, 33.20, 32.01, 25.85; HRMS (ESI) m/z [M+1]^+^: calcd for C_28_H_31_ClN_2_O, 447.2198, found, 447.2194.

#### 1-(4-((2,3-dihydro-1H-inden-5-yloxy)methyl)phenethyl)-4-(2,3-dichlorophenyl)piperazine (**22**)

White solid (ethyl acetate); Yield: 45%; Mp 77.2–78.4 ^o^C; ^1^H NMR (500 MHz, CDCl_3_) δ 7.40 (d, *J* = 7.9 Hz, 2H), 7.29 (d, *J* = 7.9 Hz, 2H), 7.20–7.14 (m, 3H), 7.01 (dd, *J* = 7.0, 2.5 Hz, 1H), 6.90 (br s, 1H), 6.80 (dd, *J* = 8.2, 2.0 Hz, 1H), 5.04 (s, 2H), 3.14 (br s, 4H), 2.93–2.83 (m, 6H), 2.83–2.66 (m, 6H), 2.14–2.06 (m, 2H);^13^C NMR (126 MHz, CDCl_3_) δ 157.82, 151.28, 145.74, 139.92, 136.46, 135.21, 134.06, 128.92, 127.73, 127.48, 124.76, 124.62, 118.64, 112.83, 110.94, 70.11, 60.41, 53.30, 51.34, 33.35, 33.20, 32.01, 29.73, 25.86; HRMS (ESI) m/z [M+1]^+^: calcd for C_28_H_30_Cl_2_N_2_O, 481.1808, found, 481.1796.

#### 1-(4-((2,3-dihydro-1H-inden-5-yloxy)methyl)phenethyl)-4-(5-chloro-2-methylphenyl)piperazine (**23**)

White solid (ethyl acetate); Yield: 72%; Mp 87.4–88.3 ^o^C; ^1^H NMR (500 MHz, CDCl_3_) δ 7.42 (d, *J* = 8.0 Hz, 2H), 7.29 (d, *J* = 8.0 Hz, 2H), 7.16 (d, *J* = 8.2 Hz, 1H), 7.13 (d, *J* = 8.0 Hz, 1H), 7.03 (d, *J* = 2.0 Hz, 1H), 7.00 (dd, *J* = 8.0, 2.0 Hz, 1H), 6.92 (br s, 1H), 6.81 (dd, *J* = 8.0, 2.0 Hz, 1H), 5.05 (s, 2H), 3.00 (t, *J* = 5.0 Hz, 4H), 2.96–2.85 (m, 6H), 2.74–2.71 (m, 6H), 2.30 (s, 3H), 2.16–2.04 (m, 2H);^13^C NMR (126 MHz, CDCl_3_) δ 157.84, 152.58, 145.75, 139.96, 136.47, 135.22, 131.97, 131.77, 130.82, 128.95, 127.75, 124.78, 122.97, 119.49, 112.85, 110.95, 70.11, 60.49, 53.57, 51.55, 33.36, 33.23, 32.04, 29.75, 25.88, 17.57; HRMS (ESI) m/z [M+1]^+^: calcd for C_29_H_33_ClN_2_O, 461.2354, found, 461.2350.

#### 1-(4-((2,3-dihydro-1H-inden-5-yloxy)methyl)phenethyl)-4-(4-bromophenyl)piperazine (**24**)

White solid (ethyl acetate); Yield: 70%; Mp 114.1–115.2 ^o^C; ^1^H NMR (500 MHz, CDCl_3_) δ 7.40 (d, *J* = 8.0 Hz, 2H), 7.38 (d, *J* = 8.2 Hz, 2H), 7.28 (d, *J* = 8.0 Hz, 2H), 7.15 (d, *J* = 8.2 Hz, 1H), 6.90 (s, 1H), 6.83 (d, *J* = 8.5 Hz, 2H), 6.80 (d, *J* = 8.2 Hz, 1H), 5.04 (s, 2H), 3.24 (t, *J* = 5.0 Hz, 4H), 2.92–2.85 (m, 6H), 2.79–2.69 (m, 6H), 2.16–2.04 (m, 2H);^13^C NMR (126 MHz, CDCl_3_) δ 157.81, 150.30, 145.75, 139.75, 136.48, 135.27, 131.89, 128.92, 127.74, 124.77, 117.66, 112.83, 111.88, 110.94, 70.09, 60.30, 53.01, 48.94, 33.23, 33.21, 32.02, 29.74.25.86; HRMS (ESI) m/z [M+1]^+^: calcd for C_28_H_31_BrN_2_O, 491.1693, found, 491.1689.

#### 1-(4-((2,3-dihydro-1H-inden-5-yloxy)methyl)phenethyl)-4-(2-(trifluoromethyl)phenyl)piperazine (**25**)

White solid (ethyl acetate); Yield: 46%; Mp 53.6–54.8 ^o^C; ^1^H NMR (500 MHz, CDCl_3_) δ 7.66 (d, *J* = 7.7 Hz, 1H), 7.55 (t, *J* = 7.7 Hz, 1H), 7.44 (d, *J* = 8.0 Hz, 1H), 7.41 (d, *J* = 7.9 Hz, 2H), 7.28 (d, *J* = 7.9 Hz, 2H), 7.26 (t, *J* = 7.7 Hz, 1H), 7.15 (d, *J* = 8.0 Hz, 1H), 6.91 (br s, 1H), 6.80 (dd, *J* = 8.1, 2.0 Hz, 1H), 5.04 (s, 2H), 3.04 (t, *J* = 5.0 Hz, 4H), 2.96–2.83 (m, 6H), 2.72 (m, 6H), 2.15–2.05 (m, 2H);^13^C NMR (126 MHz, CDCl_3_) δ 157.84, 152.61, 145.74, 140.00, 136.45, 135.19, 132.76, 128.93, 127.73, 127.24, 127.19, 124.76, 124.05, 112.85, 110.95, 70.12, 60.52, 53.57, 53.45, 33.38, 33.21, 32.02, 25.86; HRMS (ESI) m/z [M+1]^+^: calcd for C_29_H_31_F_3_N_2_O, 481.2461, found, 481.2458.

#### 1-(4-((2,3-dihydro-1H-inden-5-yloxy)methyl)phenethyl)-4-(4-(trifluoromethyl)phenyl)piperazine (**26**)

White solid (ethyl acetate); Yield: 42%; Mp 135.3–136.5 ^o^C; ^1^H NMR (500 MHz, CDCl_3_) δ 7.52 (d, *J* = 8.6 Hz, 2H), 7.40 (d, *J* = 7.9 Hz, 2H), 7.28 (d, *J* = 7.9 Hz, 2H), 7.14 (d, *J* = 8.2 Hz, 1H), 6.96 (d, *J* = 8.6 Hz, 2H), 6.90 (br s, 1H), 6.79 (dd, *J* = 8.2, 2.0 Hz, 1H), 5.04 (s, 2H), 3.35 (t, *J* = 5.0 Hz, 4H), 2.93–2.85 (m, 6H), 2.77–2.65 (m, 6H), 2.15–2.05 (m, 2H);^13^C NMR (126 MHz, CDCl_3_) δ 157.81, 153.28, 145.75, 139.72, 136.49, 135.30, 128.90, 127.73, 126.42, 126.39, 124.76, 114.54, 112.83, 110.94, 70.10, 60.28, 52.91, 47.96, 33.25, 33.20, 32.00, 29.71, 25.84; HRMS (ESI) m/z [M+1]^+^: calcd for C_29_H_31_F_3_N_2_O, 481.2461, found, 481.2454.

### Biological evaluation

#### Assay of antiproliferative activity

The antiproliferative activity of compounds **5**–**26** was assessed using the CCK-8 assay (Chen et al., 2016; Chen et al., 2017; Chen et al., 2018; Han et al., 2020; Guo et al., 2021; Hu et al., 2022; Zhou et al., 2022). The 96-well plates (1×10^5^ cells/mL) were seeded with cells in a medium, the plates were cultured at 37 °C for 24 h, then different concentrations of tested drugs were added, and the plates were cultured for 24 h. After 24 h, 10 μL of CCK-8 solution (5 mg/ml) was added to the wells, and the cells were cultured for 1 h at 37°C. Cell growth inhibition was performed by measuring the absorbance at 450 nm using a microplate reader, and the percentage of cell growth inhibition was then calculated for each tested drug.

### Antagonistic activity in a_1_-ARs by dual-luciferase reporter gene assay

The AR antagonist effect of tested compounds was evaluated using the luciferase reporter gene assay (Xu et al., 2014; Xu et al., 2015; Zuo et al., 2017; Xu et al., 2018). Briefly, RLUs were used to indicate firefly and Renilla luciferase activity, and the activities were evaluated using dual luciferase assay kits (Promega) in accordance with the manufacturer’s instructions. RLUs were measured using a GloMaxTM 96-Microplate Luminometer (Promega) and three individual experiments were performed as the mean ± SEM. For agonists, fold of induction = LU_induced_/RLU_uninduced_. For antagonists, % of control = 100 × RLU (agonist + antagonist)/RLU (agonist alone). All RLUs were normalized according to firefly RLUs/Renilla RLUs. EC_50_/IC_50_ values were expressed as μM, and Graph-pad Prism 5 software was used to calculate the IC_50_ of phenylephrine (μM) by plotting the data using nonlinear regression analysis.

### Fluorescence polarization

The binding of the derivatives **5**, **8**, **12**, **14**, **15**, **19**, **21**, **22**, **25**, **26** and enzalutamide to the AR was analyzed by FP technique using the PolarScreen™ AR Competitor Assay according to the manufacturer’s instructions (Chen et al., 2019a; Chen et al., 2019b). Briefly, titrations are performed between the tested compounds and the preformed Fluormone™AL Green and the AR-LBD (GST) complex. The tested mixture was allowed to equilibrate at room temperature in 384-well plates for 4 h. Then, the fluorescence polarization values of the tested mixture were performed using a SpectraMax®Paradigm^®^ Multi-Mode Detection Platform at 485 nm (excitation wavelength) and 535 nm (emission wavelength). Data of the ligand binding assays were analyzed using Prism software (GraphPad Software, Inc.).

### Molecular docking simulation

The crystal structure file (PDB code: 2OZ7) of the complex was downloaded from the protein crystal structure database (Protein Data Bank, http://www.rcsb.org/pdb/) as the basis for molecular docking. Molecular docking was performed using the Surflex-Dock module in SYBYL-X2.0 and the program package in AutoDock (Chen et al., 2019a; Chen et al., 2019b). The X-ray crystallographic structures of androgen receptors were obtained from the RCSB Protein Data Bank (http://www.rcsb.org/). A box of 40 × 40 × 30 Å3 around the binding site was built and grid spacing is 1 Å whose center was considered the geometric center of the ligand. Finally, the representative compounds and the exogenous ligands with 3D structure were docked into the binding cavity of AR to obtain the lowest energy docking method from 10 docking modes given by cluster analysis.

## Results and discussions

### Chemistry

Arylpiperazine derivatives **5–26** were synthesized using **1** as starting material (Scheme 1). Firstly compound **1** is reduced by borane–methyl sulfide complex (2 M in tetrahydrofuran) to obtain compound **2**. The obtained crude products **2** were directly used in the next step without purification. Then, compound **3** was obtained after treatment of compound **2** and 2,3-dihydro-1H-inden-5-ol in the presence of potassium carbonate in acetonitrile. Subsequently, compound **4** was achieved by treatment of compound **3** with 4-toluene-ulfonyl chloride in the presence of triethylamine and 4-dimethylaminopyridine in methylene chloride. Finally, compound **4** was treated with the different arylpiperazines in the presence of potassium carbonate in acetonitrile to obtain arylpiperazine derivatives **5**–**26** (Yield: 40–90%). All synthesized derivatives were confirmed by ^1^H NMR, ^13^C NMR, and HRMS.

### SAR analysis for antiproliferative and AR antagonist assay

Antiproliferative activity of the novel arylpiperazine derivatives was firstly evaluated against LNCaP cells using the CCK-8 assay. Then, PC-3 cells were used to determine whether the arylpiperazine derivatives depended on AR to exhibit inhibitory activity and potent cytotoxic activities. Naftopidil and finasteride (Banday et al., 2014) were used as control compounds. RWPE-1 cells were used to compare their toxicity. To clarify whether the antiproliferative activity was related to any interference with AR function, the AR antagonist effect was evaluated using the luciferase reporter gene assay. In order to determine whether anti-proliferation activities of derivatives were related to the interference of AR function, the AR antagonist effect of tested compounds was evaluated using the luciferase reporter gene assay. The AR luciferase assays were conducted under the co-treatment of 1 nM AR agonist R1881, and the antagonistic activity was measured by inhibiting the R1881-induced luciferase expression.

As shown in Table 1, the derivatives **5**, **8**, **12**, **15**, **19**, **21**, **22**, **25**, and **26** exhibited strong cytotoxic activities against LNCaP cells (IC_50_ <10 μM). Compared to arylpiperazine derivatives (Chen et al., 2016; Chen et al., 2017; Chen et al., 2019a), some derivatives exhibited potent cytotoxic activities against LNCaP cells. In addition, the majority of derivatives exhibited higher anticancer activity than reported arylpiperazine derivatives (Chen et al., 2016; Chen et al., 2017; Chen et al., 2018; Chen et al., 2019b) against PC-3 cells. Moreover, those derivatives displayed relatively strong AR-antagonistic potency (>55% inhibition), which exhibited higher antagonistic potency than previously reported derivatives (Chen et al., 2019a; <50% inhibition). Meanwhile, those derivatives displayed comparable antagonistic activity to ABO analogs containing the piperazine moiety (Chen et al., 2019b). But, compared to ABO analogs, the derivatives **5**, **21**, **25**, and **26** exhibited strong antagonistic potency against AR (>60% inhibition). Especially, derivative **21** demonstrated the highest antagonistic potency (76.2% inhibition). The derivatives **14** and **15** had relatively strong antagonistic potency against AR (>50% inhibition), consistent with the LNCaP cells’ antiproliferation activity (IC_50_ <10 μM). However, they also exhibited toxicity against PC-3 cells. Moreover, **11**, **17**, **18**, **20**, **23**, and **24** displayed weak cytotoxic activities against LNCaP cells and weak antagonistic potency against AR. However, they displayed strong cytotoxic activities against PC-3 cells and exhibited low cytotoxic character toward RWPE-1 cells.

**TABLE 1:** Antiproliferative and AR antagonist activity of derivatives **5**–**26**.

Compound	PC-3^a^ IC50 (μM)^b^	LNCaP^a^ IC50 (μM)^b^	RWPE-1^a^ IC50 (μM)^b^	AR antagonistic activity % (10 μM)^c^
**5**	>50	3.67 ± 0.14	>50	62.5 ± 1.2
**6**	11.34 ± 0.15	23.45 ± 0.12	11.58 ± 0.21	N.D
**7**	>50	15.29 ± 0.13	7.68 ± 0.15	N.D
**8**	>50	7.37 ± 0.15	>50	58.1 ± 0.8
**9**	>50	42.68 ± 0.14	10.96 ± 0.16	N.D
**10**	17.23 ± 0.21	20.87 ± 0.22	9.24 ± 0.23	N.D
**11**	4.31 ± 0.13	>50	>50	35.2 ± 1.1
**12**	25.62 ± 0.17	8.12 ± 0.11	>50	56.3 ± 1.2
**13**	>50	>50	>50	N.D
**14**	0.87 ± 0.12	3.09 ± 0.11	>50	56.2 ± 0.7
**15**	8.94 ± 0.21	9.72 ± 0.13	21.69 ± 0.23	54.4 ± 0.6
**16**	>50	18.56 ± 0.17	>50	N.D
**17**	1.45 ± 0.17	17.92 ± 0.13	>50	43.3 ± 0.7
**18**	1.92 ± 0.24	10.69 ± 0.14	>50	44.2 ± 0.5
**19**	>50	6.87 ± 0.06	32.67 ± 0.14	59.2 ± 0.9
**20**	1.56 ± 0.14	16.98 ± 0.15	>50	45.2 ± 0.3
**21**	>50	3.74 ± 0.24	>50	76.2 ± 0.4
**22**	23.48 ± 0.23	6.32 ± 0.14	>50	57.0 ± 1.2
**23**	0.95 ± 0.14	12.56 ± 0.22	>50	40.1 ± 0.8
**24**	2.31 ± 0.23	14.73 ± 0.15	>50	42.7 ± 1.1
**25**	>50	1.24 ± 0.15	>50	68.4 ± 0.5
**26**	>50	2.57 ± 0.24	>50	65.3 ± 1.2
**Naftopidil**	42.10 ± 0.79	22.36 ± 0.61	>50	N.D
**Finasteride**	17.80	13.53	N.D	N.D
**R1881**	N.D	N.D	N.D	N.E
**Enzalutamide**	N.D	N.D	N.D	84.7 ± 1.4

N.D, not determined.

N.E, no antagonistic effect.

a PC-3, and LNCaP, human prostate cancer cell line; RWPE-1, normal non-cancer human prostate epithelial cell line.

b IC_50_ values were the mean ± standard deviation of the three experiments.

c Inhibition rate was shown as a ratio to the R1881 control.

The SAR of the arylpiperazine derivatives was fully explored and discussed. Taking compound **5** as a lead compound, (1) first, compared with **6** and **7**, strong cytotoxic activity was displayed by **5** against LNCaP cells (IC_50_ = 3.67 μM), and it had potent antagonistic potency against AR (62.5% inhibition). These results suggested that other aryl groups substituted at the 4-position of the piperazine ring were inauspicious for improved activity. However, estrone derivatives containing the piperazine moiety **6** (IC_50_ = 1.42 μM) displayed strong cytotoxic activities against LNCaP cells (Chen et al., 2018). (2) The substitute’s position and type on the phenyl group also affected the biological activities. Compared with **9** (3-CH_3_) and **10** (4-CH_3_), **8** (2-CH_3_) displayed strong cytotoxic activity against LNCaP cells (IC_50_ = 7.37 μM) and relatively strong antagonistic potency against AR (58.1% inhibition). Meanwhile, estrone piperazine derivatives (2-CH_3;_ IC_50_ = 0.83 μM) displayed strong cytotoxic activity against LNCaP cells (Chen et al., 2018). However, other derivatives with a methyl substituent on the phenyl group exhibited weak cytotoxic activity against the tested cells (Chen et al., 2017; Chen et al., 2018). The activity profiles indicated that the *o*-substituted phenyl group derivative (2-CH_3_) was beneficial for cytotoxic activity and antagonistic activity. (3) Compared with compounds **8** and **9**, relatively strong cytotoxic activities were exhibited by **11** (2,5-2CH_3_) against PC-3 cells. Meanwhile, estrone piperazine derivative (2,5-2CH_3_) also displayed strong cytotoxic activity against PC-3 cells (IC_50_ = 3.41 μM). These results suggested that the disubstituted methyl derivative was beneficial for cytotoxic activities. (4) Methoxyl-substituted derivatives **12** (2-OCH_3_), **14** (3-OCH_3_), and **15** (4-OCH_3_) had strong cytotoxic activities against LNCaP cells and relatively strong antagonistic potency against AR. But, methoxyl-substituted derivatives exhibited weak or no cytotoxic activity LNCaP cells except the derivative **11** (Chen et al., 2017; Chen et al., 2018). Moreover, the *m*-substituted phenyl group derivative (**14**) and *p*-substituted phenyl group derivative (**15**) also exhibited toxicity against PC-3 cells (Iwasa et al., 2007; Dreaden et al., 2012; George et al., 2018; Saito et al., 2018), and methoxyl-substituted derivatives also exhibited toxicity against PC-3 cells except the derivative **12** (Chen et al., 2017; Chen et al., 2018). (5) Compared with **16**, strong cytotoxic activity was displayed by **17** and **18** against PC-3 cells. However, fluro-substituted derivatives displayed weak cytotoxic activity against PC-3 cells (Chen et al., 2017; Chen et al., 2018). Moreover, these compounds displayed relatively weak antagonistic potency against AR (<50% inhibition). These findings indicated that fluro-substituted phenyl group derivatives were inauspicious for improved activity. (6) Strong cytotoxic activities were displayed by **19** (2-Cl) and **21** (4-Cl) against LNCaP cells, and they had relatively strong antagonistic potency against AR and weak cytotoxic activity against PC-3 cells. The derivative **20** (3-Cl) displayed relatively weak antagonistic potency against AR and strong toxicity against PC-3 cells. However, **14** (2-Cl) and **16** (4-Cl) displayed weak cytotoxic activity against PC-3 and LNCaP cells, and 15 (3-Cl) displayed strong cytotoxic activity against PC-3 cells (Chen et al., 2017). In addition, in Chen et al. (2018), fluro-substituted derivatives displayed strong cytotoxic activities against LNCaP cells except **18** and exhibited weak cytotoxic activity against PC-3 cells. (6) More effective cytotoxic activity was displayed by **23** (2-CH_3_, 5-Cl, IC_50_ = 0.95 μM) than **8** (2-CH_3_) and **20** (3-Cl) against PC-3 cells. Activity profiles indicated that the introduction of methyl and chloro into the phenyl group was beneficial for cytotoxic activity. (7) The trifluoromethyl-substituted derivatives **25** and **26** exhibited strong cytotoxic activities against LNCaP cells (IC_50_ <3 μM) and relatively strong antagonistic potency against AR (>65% inhibition). These derivatives also exhibited weak inhibitory activity toward PC-3 cells and normal human prostate epithelial cells (RWPE-1). However, in Chen et al. (2018) and Chen et al. (2017), the trifluoromethyl-substituted derivatives displayed weak cytotoxic activities against PC-3 and LNCaP cells except **21** against PC-3.

Taken together, compared to the reported derivatives (Chen et al., 2019a; Chen et al., 2019b), the SAR studies indicated that the *o*-substituted phenyl group derivatives displayed moderate to strong cytotoxic activities against LNCaP cells and relatively strong antagonistic potency against AR. These derivatives also exhibited weak inhibitory activity toward PC-3 cells, suggesting that these compounds depended on AR to exert inhibitory activity. The abovementioned results can also lead to a tool that can further design these derivatives as AR antagonists for *in vitro* and *in vivo* studies.

### Binding affinity assay of arylpiperazine derivatives with potent AR antagonistic potency

To further study the binding affinity of the arylpiperazine derivatives with potent AR antagonistic potency, based on the fluorescent tracer and nonfluorescent antagonist competing for binding to AR, the binding affinity of **5**, **8**, **12**, **14**, **15**, **19**, **21**, **22**, **25**, and **26** to AR was examined using the binding assay by the fluorescence polarization technique. The results are shown in Table 2; the tested arylpiperazine derivatives exhibited strong AR-binding affinities (IC_50_ <3 μM), and the majority of derivatives possessed higher binding affinities than some reported derivatives (Chen et al., 2019a; Chen et al., 2019b). Among these derivatives, the derivative **21** (IC_50_ = 0.65 μM) demonstrated the highest binding affinity to the AR, possessing higher binding affinities than enzalutamide (IC_50_ = 1.32 μM). Among all tested derivatives, a correlation can be identified between the effect on antagonistic activity and AR-binding affinity. For example, **5, 21, 25**, and **26** with higher affinity for AR (IC_50_ <2 μM) also showed relatively strong antagonistic activity (>60% inhibition). These results indicated that the AR-binding affinity may play a key role in promoting the AR antagonistic activity, and these arylpiperazine derivatives may be efficient AR antagonists for PCa treatment. So, derivative **21** was selected to further investigate the binding site to the AR.

**TABLE 2:** Binding affinity of **5**, **8**, **12**, **14**, **15**, **19**, **21**, **22**, **25**, and **26** to mutant AR.

Compound	IC_50_/µm^a^
**5**	1.46 ± 0.24
**8**	2.35 ± 0.09
**12**	2.53 ± 0.15
**14**	2.62 ± 0.25
**15**	2.85 ± 0.65
**19**	2.06 ± 0.21
**21**	0.65 ± 0.08
**22**	2.47 ± 0.11
**25**	1.22 ± 0.15
**26**	1.43 ± 0.18
Enzalutamide	1.32 ± 0.78

a The data represent the mean ± standard deviation of the three experiments.

### Docking study

To further understand the binding sites (ligand binding pocket (LBP), activation function-2 (AF2), and binding function 3 (BF3)) of the derivative **21** and AR, and to explore their dominant interactions with AR, the docking experiment was carried out using SYBYL and AutoDock software (Axerio-Cilies et al., 2011; Lack et al., 2011). The results were summarized in Table 3.

**TABLE 3:** The binding affinities (kcal/mol) of docking of derivative **21** with three binding sites of AR.

Binding site	Compound 21
LBP (PDB ID: 2OZ7)	−10.8
AF2 (PDB ID: 2YHD)	−5.5
BF3 (PDB ID: 2YLO)	−5.6

**FIGURE 2:**
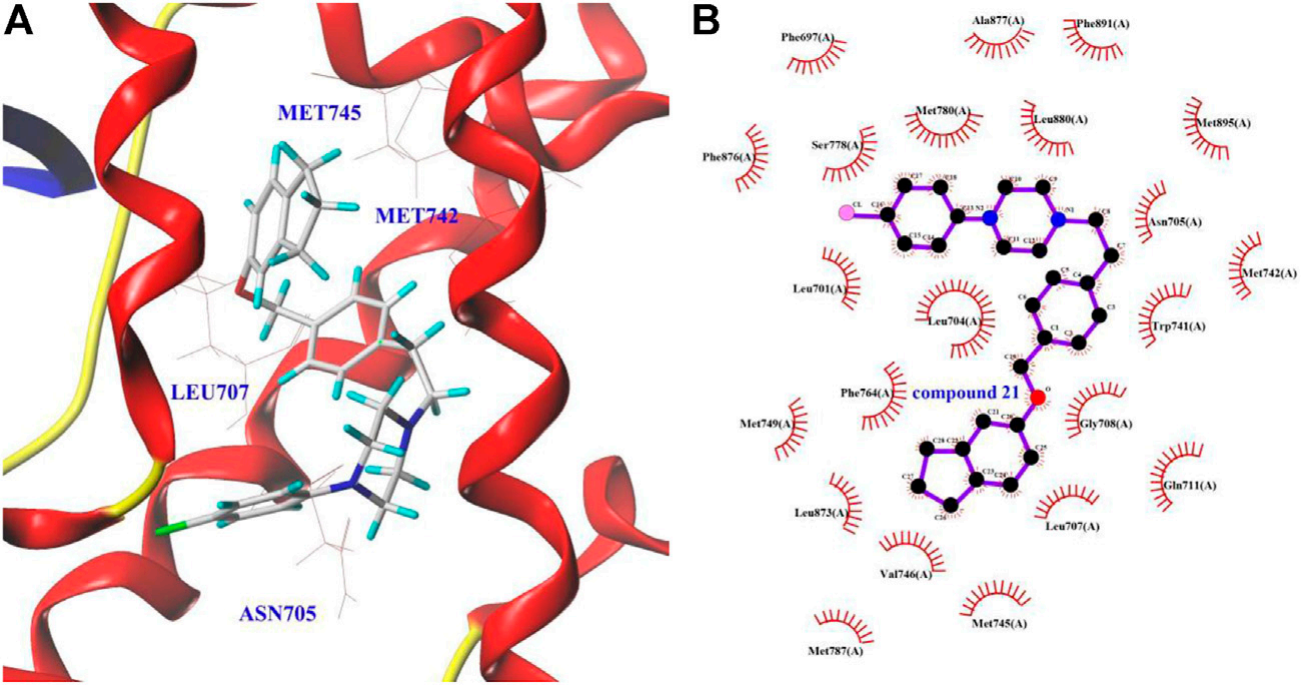
The docking view of compound **21**–AR interaction.

As shown in Table 3, the binding free energy of derivative **21** with LBP was −10.8 kcal/mol. However, the binding free energy of it with AF2 and BF3 was between −5.5 and −5.6 kcal/mol, respectively. The results suggested that the AR-LBP site was the major binding site for derivative **21**. It can be observed in Figure 2 that the binding of derivative **21** to the AR-LBP site mainly through the hydrophobic interactions with Asn705, Met742, Met745, Leu707, etc. However, in Chen et al. (2019a), a molecular docking study suggested that derivative **16** mainly binds to the AR-LBP site by hydrogen bonding interactions, and in Chen et al. (2019b), ABO piperazine analogs **23** mainly bind to the AR-LBP site through the formation of Van der Waals force with amino acid residues.

## Conclusion

In this work, 22 novel arylpiperazine derivatives were designed and synthesized, and their biological evaluation and molecular docking were reported. The derivatives **5**, **8**, **12**, **19**, **21**, **22**, **25**, and **26** exhibited strong cytotoxic activities against LNCaP cells *versus* PC-3 cells, and those derivatives displayed relatively strong AR antagonistic potency (>55% inhibition) and excellent AR-binding affinities (IC_50_ <3 μM), which exhibited higher bioactivity than previously reported arylpiperazine derivatives. Among them, derivative **21** exhibited the highest binding affinity for AR (IC_50_ = 0.65 μM) and the highest antagonistic potency (76.2% inhibition). A molecular docking study suggested the binding of derivative **21** to the AR-LBP site mainly occurred through the hydrophobic interactions with amino acid residues. The SAR studies suggested that the *o*-substituted phenyl group derivatives exhibited relatively improved bioactivity. These results can lead to a tool that can further design these novel derivatives as AR antagonists for *in vitro* and *in vivo* studies.

## Data Availability

The original contributions presented in the study are included in the article/Supplementary Material; further inquiries can be directed to the corresponding authors.
