# Supplementary material for: Synthesis, bioactivity, and molecular docking of novel arylpiperazine derivatives as potential AR antagonists
Source: Front Chem. 2022 Aug 15;10:947065. doi: 10.3389/fchem.2022.947065 (PMC9420858; doi:10.3389/fchem.2022.947065)
Supplement: Supplementary file 1 [file DataSheet1.docx]

**Supporting information**

**Synthesis, Bioactivity and Molecular Docking of Novel Arylpiperazine Derivatives as** **Potential AR Antagonists**

**Yueheng Qi^1,2^, Hong Chen^2^**^^[[1]](#footnote-0)^*^**, Shijin Chen^2^, Jianliang Shen^3,^**^^[[2]](#footnote-1)^*^**, Jingguo Li^1^**^^[[3]](#footnote-2)^*^

*^1^ Henan Provincial People's Hospital, People's Hospital of Zhengzhou University, Zhengzhou, Henan 450003, PR China*

*^2^ Luoyang Key Laboratory of Organic Functional Molecules, College of Food and Drug, Luoyang Normal University, Luoyang, Henan 471934, PR China*

*^3^ School of Ophthalmology & Optometry, School of Biomedical Engineering, Wenzhou Medical University, Wenzhou, Zhejiang 325035, PR China*

Corresponding author E-mail: [chenwepo@sina.com;](mailto:chenwepo@sina.com;) [shenjl@wiucas.ac.cn](mailto:liziyong@mails.ccnu.edu.cn); lijingguo@zzu.edu.cn

**^1^H NMR spectrum, ^13^C NMR spectrum and HRMS spectrum of compounds**

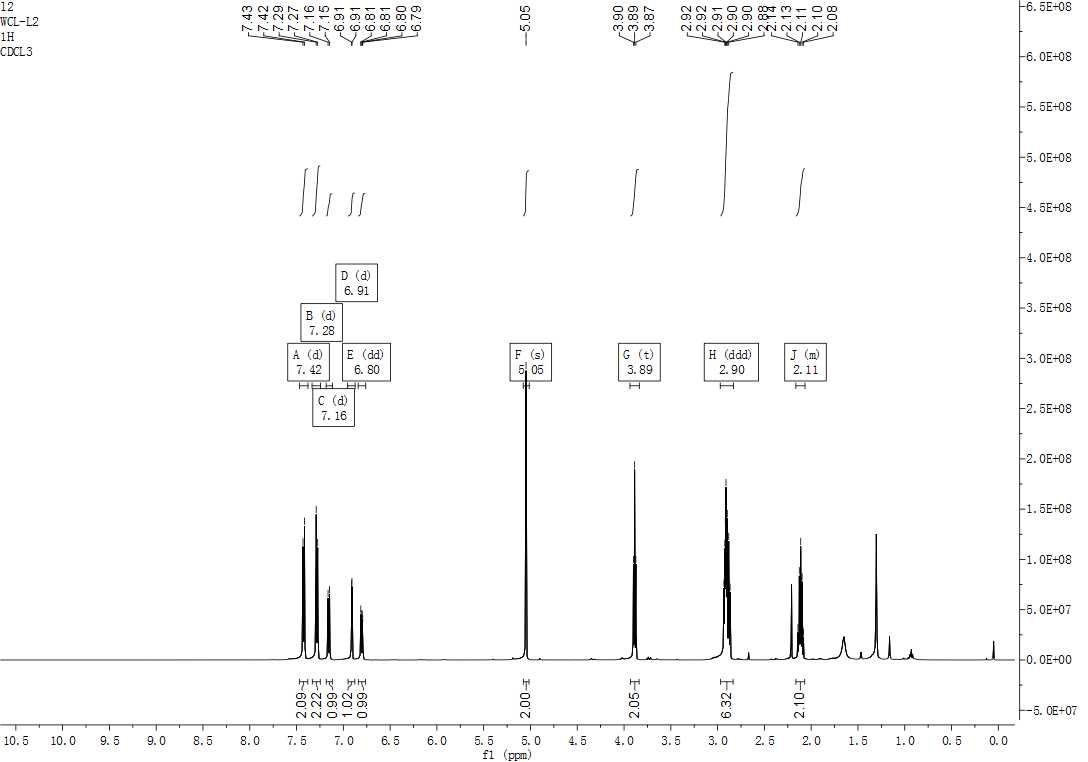


^1^H NMR spectrum of compound **3**

HRMS of compound **3**

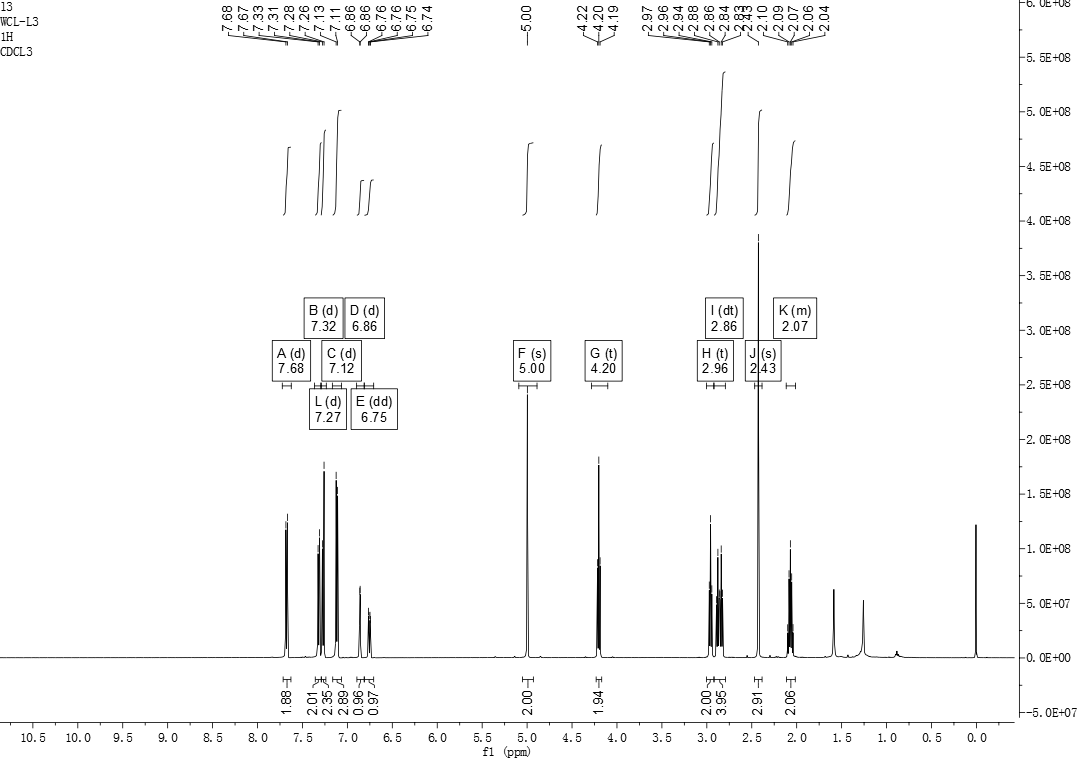


^1^H NMR spectrum of compound **4**

HRMS of compound **4**

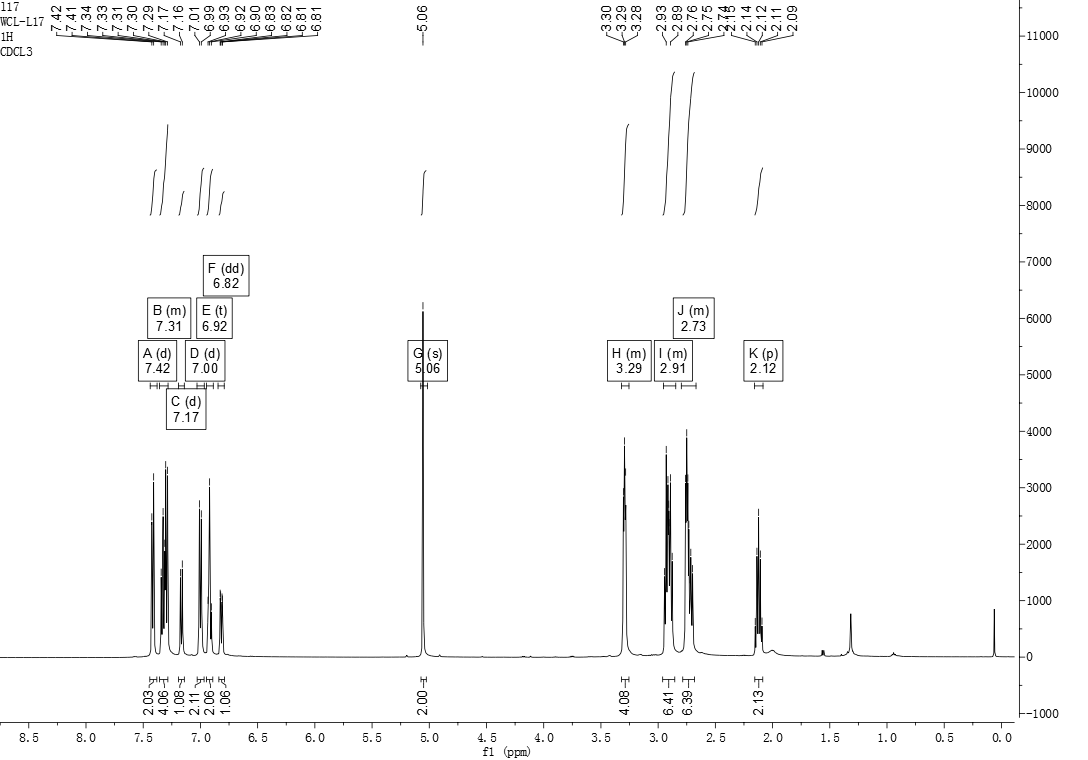


^1^H NMR spectrum of compound **5**


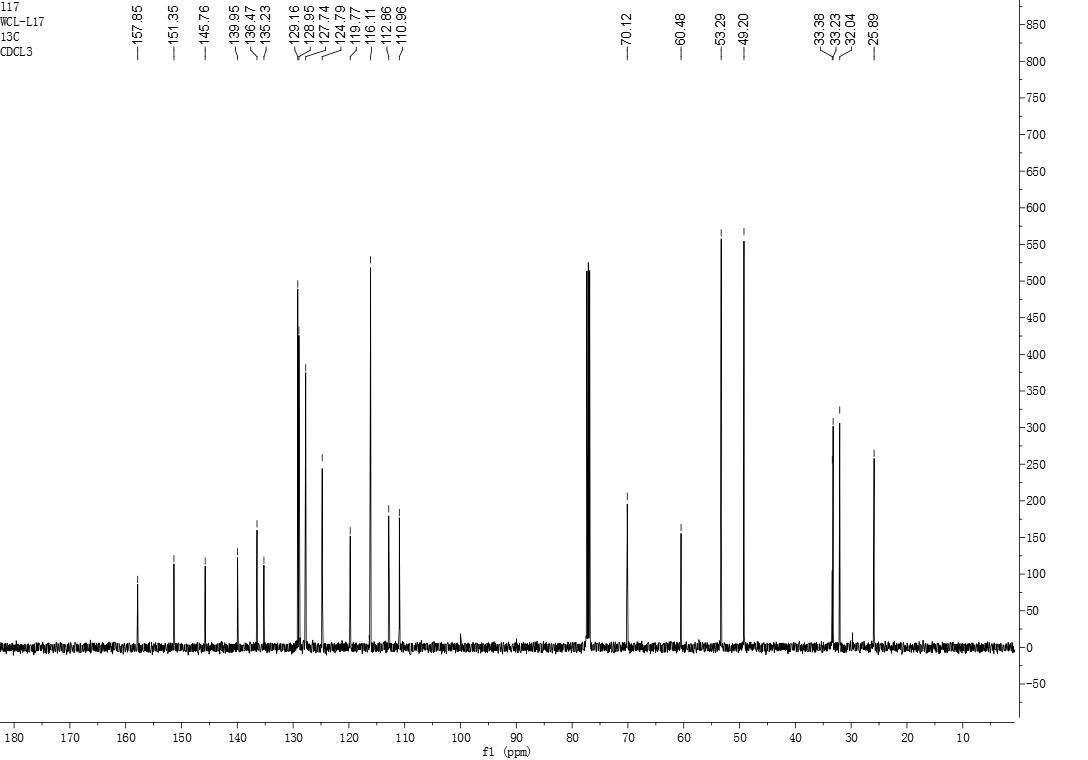


^13^C NMR spectrum of compound **5**

HRMS of compound **5**

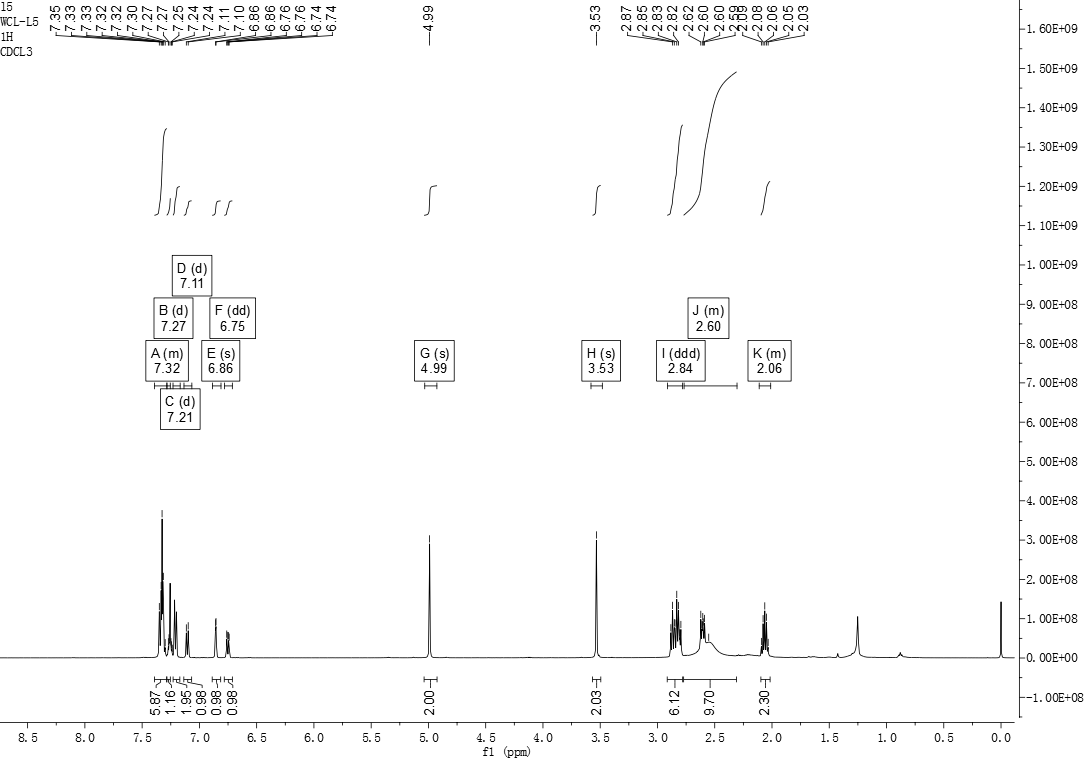


^1^H NMR spectrum of compound **6**


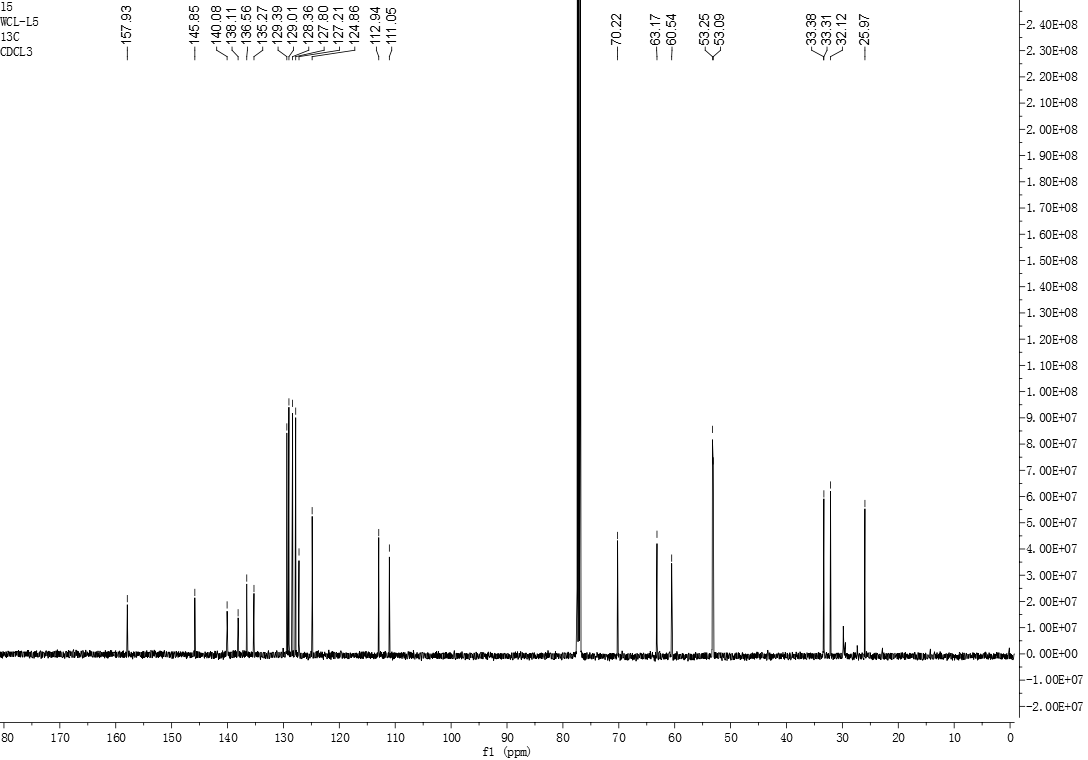


^13^C NMR spectrum of compound **6**

HRMS of compound **6**

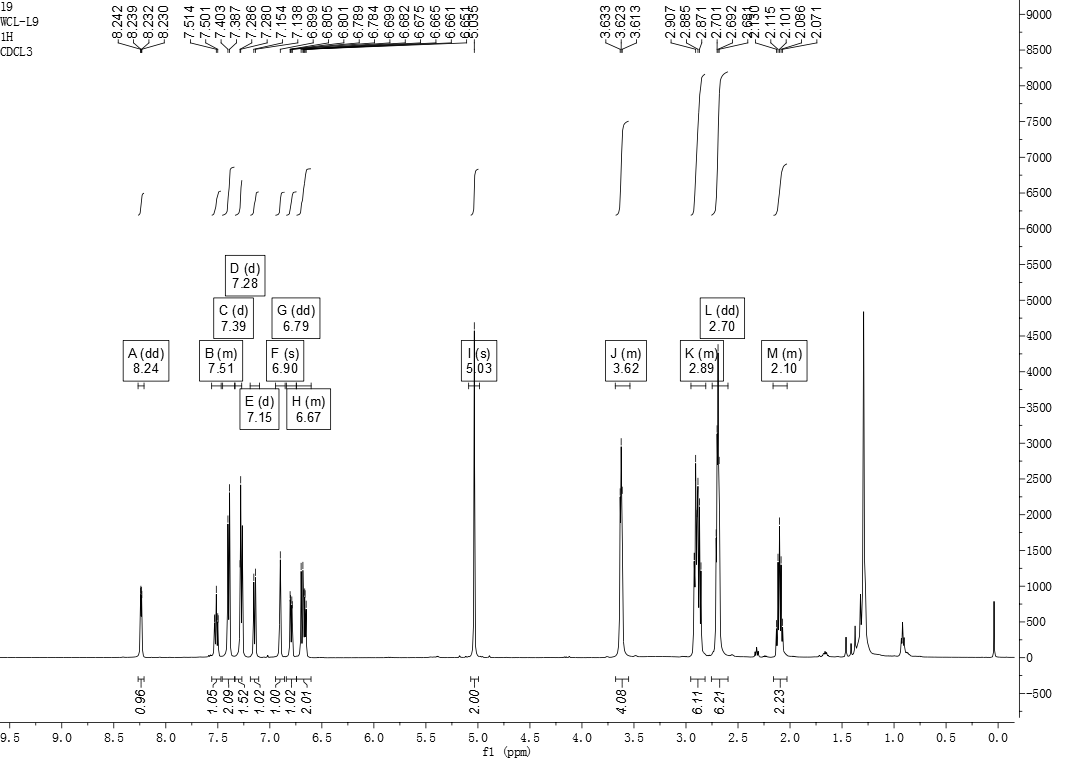


^1^H NMR spectrum of compound **7**


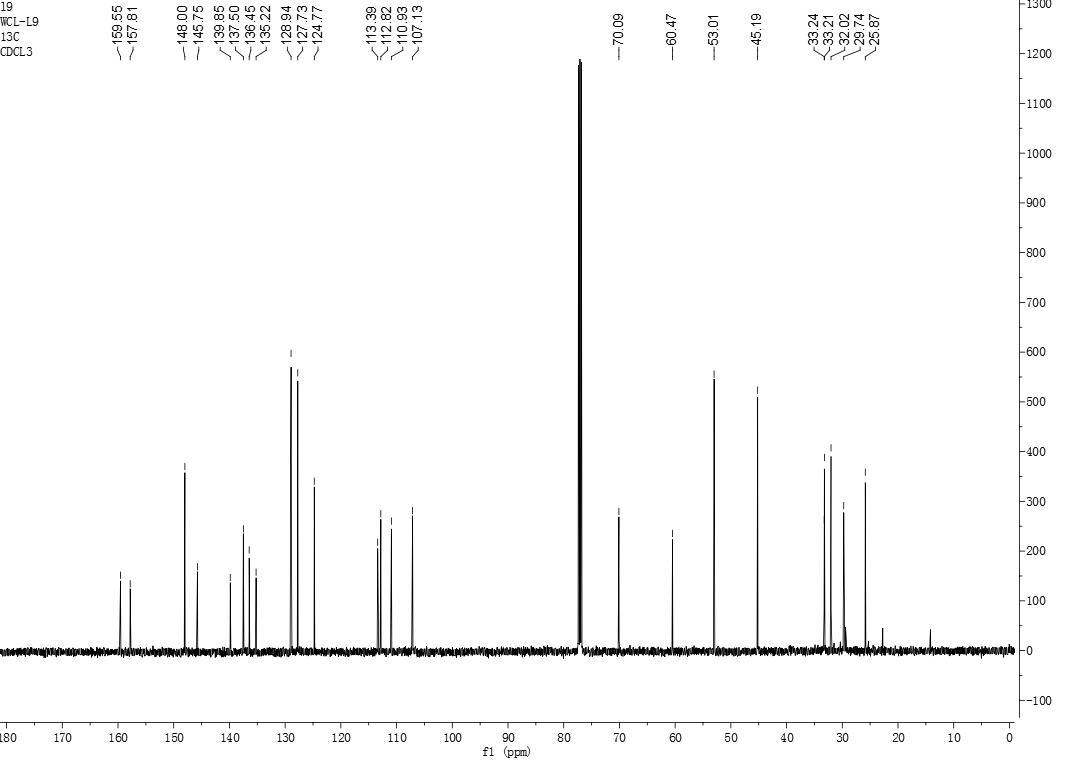


^13^C NMR spectrum of compound **7**

HRMS of compound **7**

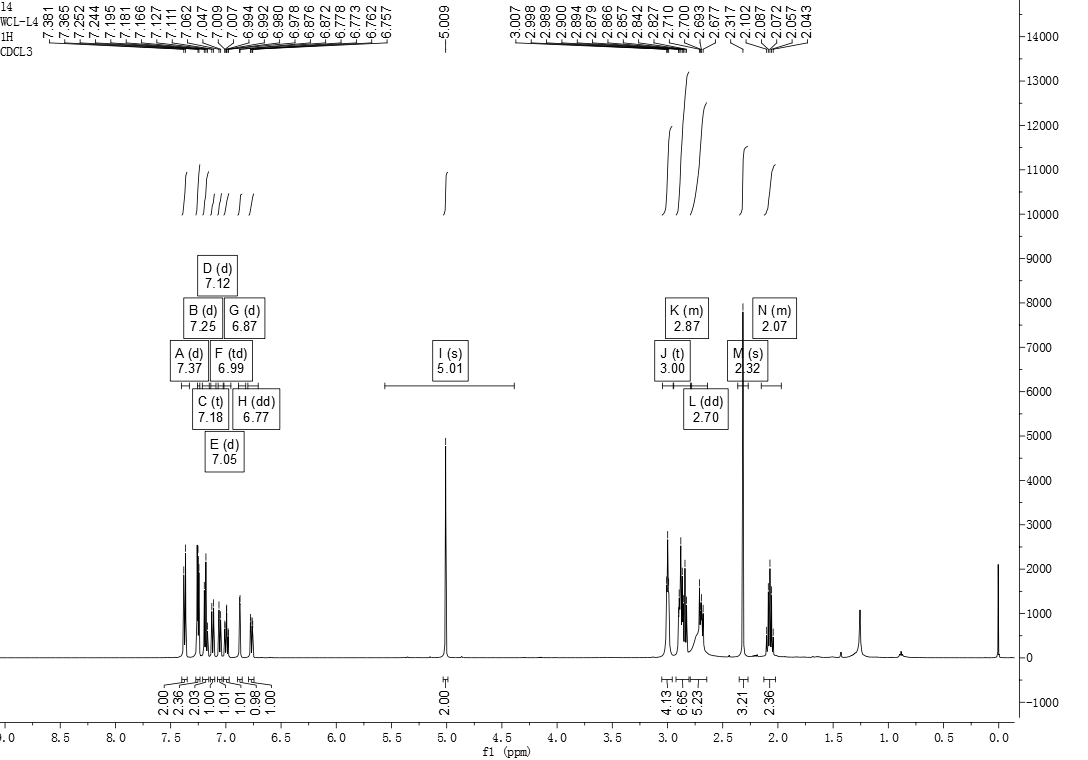


^1^H NMR spectrum of compound **8**


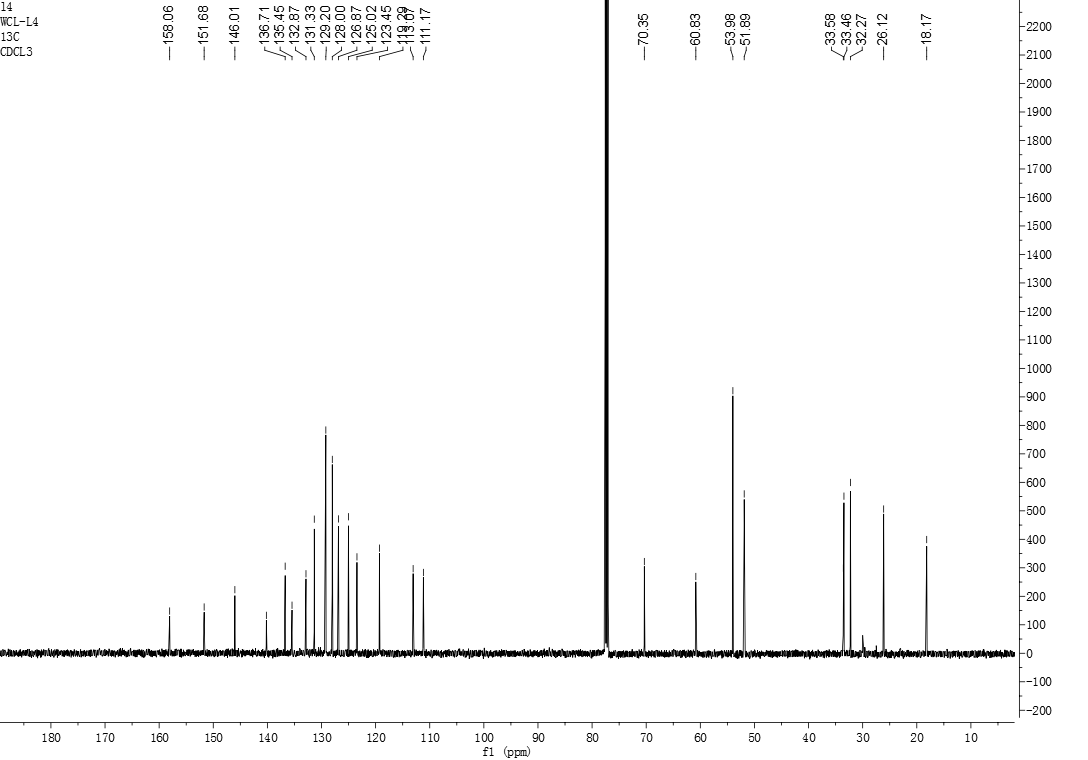


^13^C NMR spectrum of compound **8**

HRMS of compound **8**

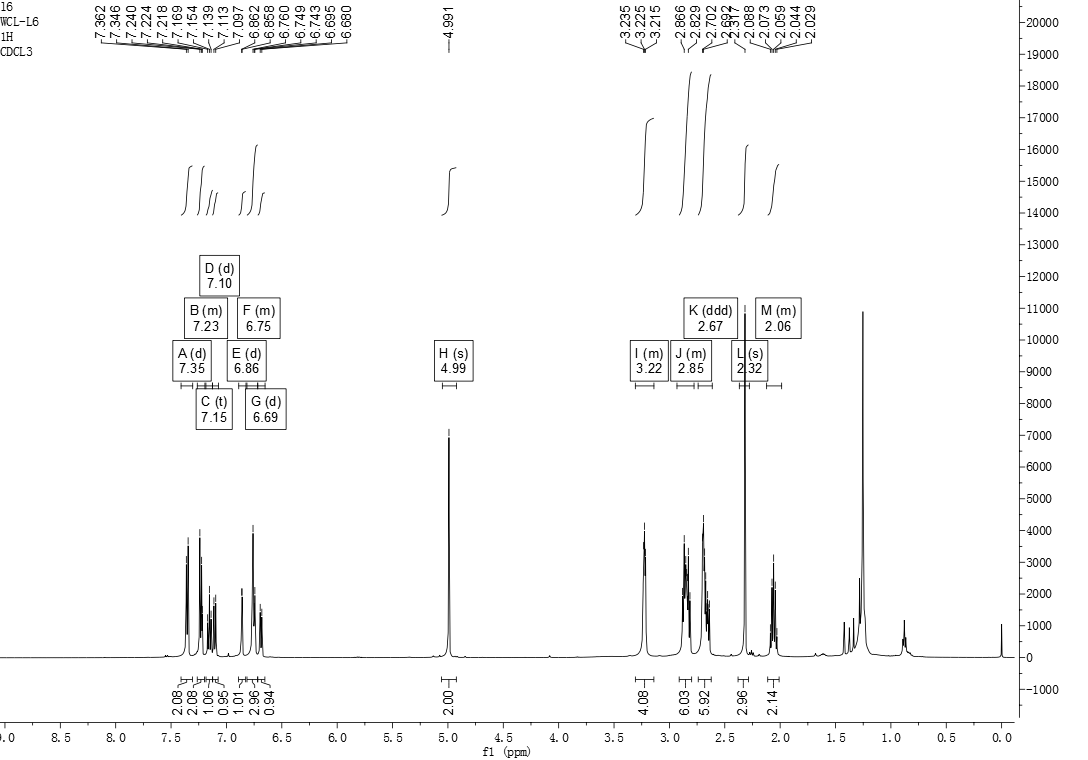


^1^H NMR spectrum of compound **9**


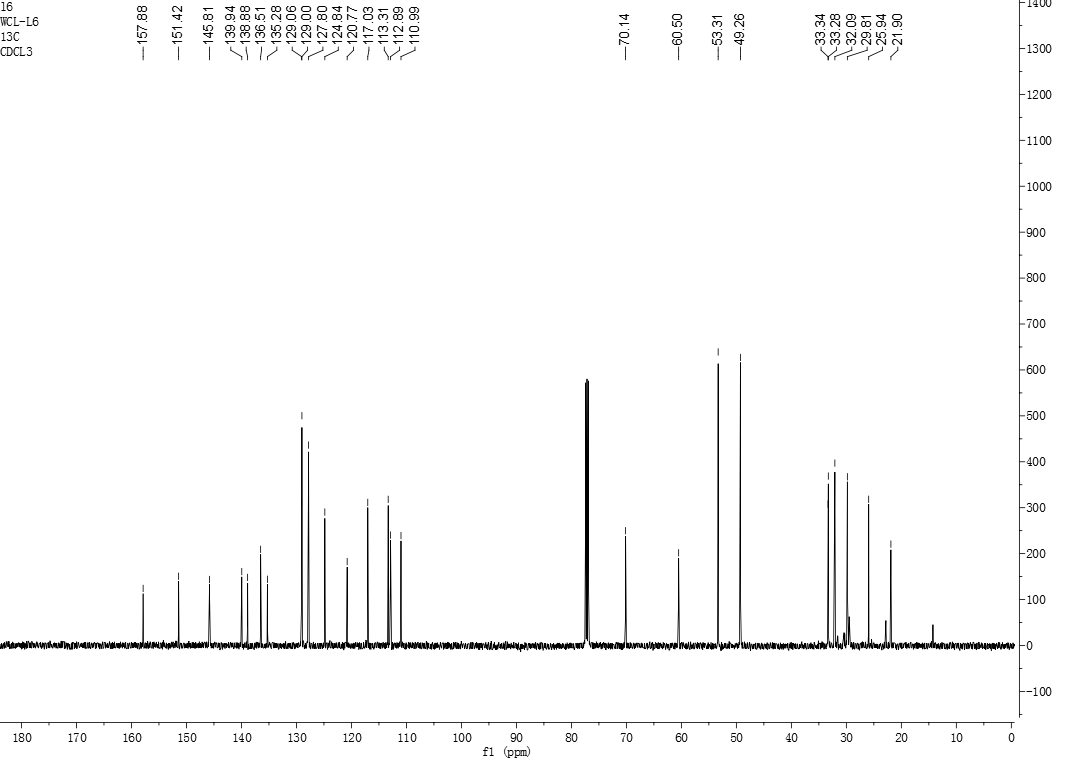


^13^C NMR spectrum of compound **9**

HRMS of compound **9**

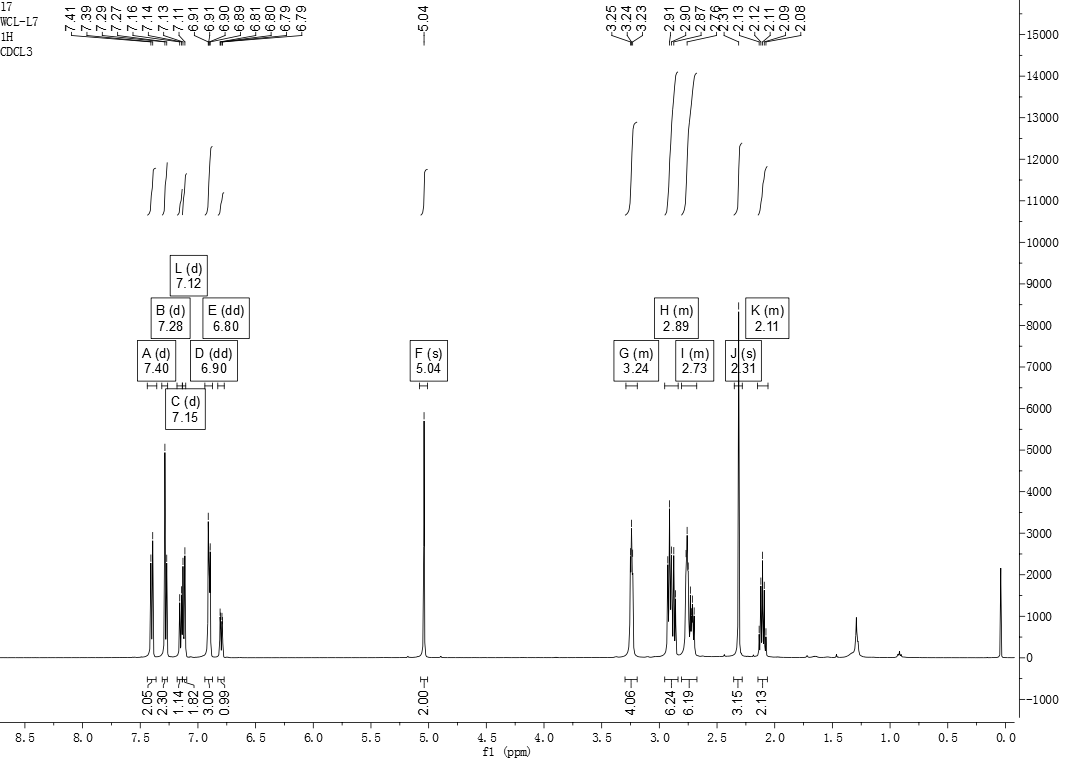


^1^H NMR spectrum of compound **10**


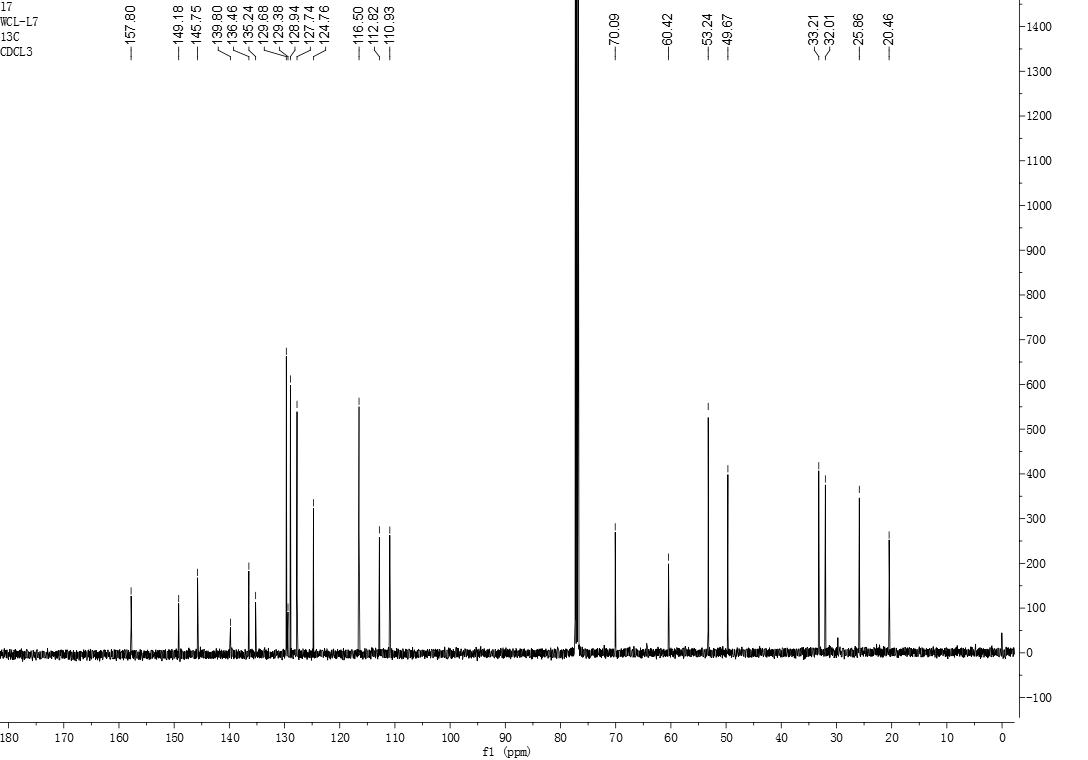


^13^C NMR spectrum of compound **10**

HRMS of compound **10**

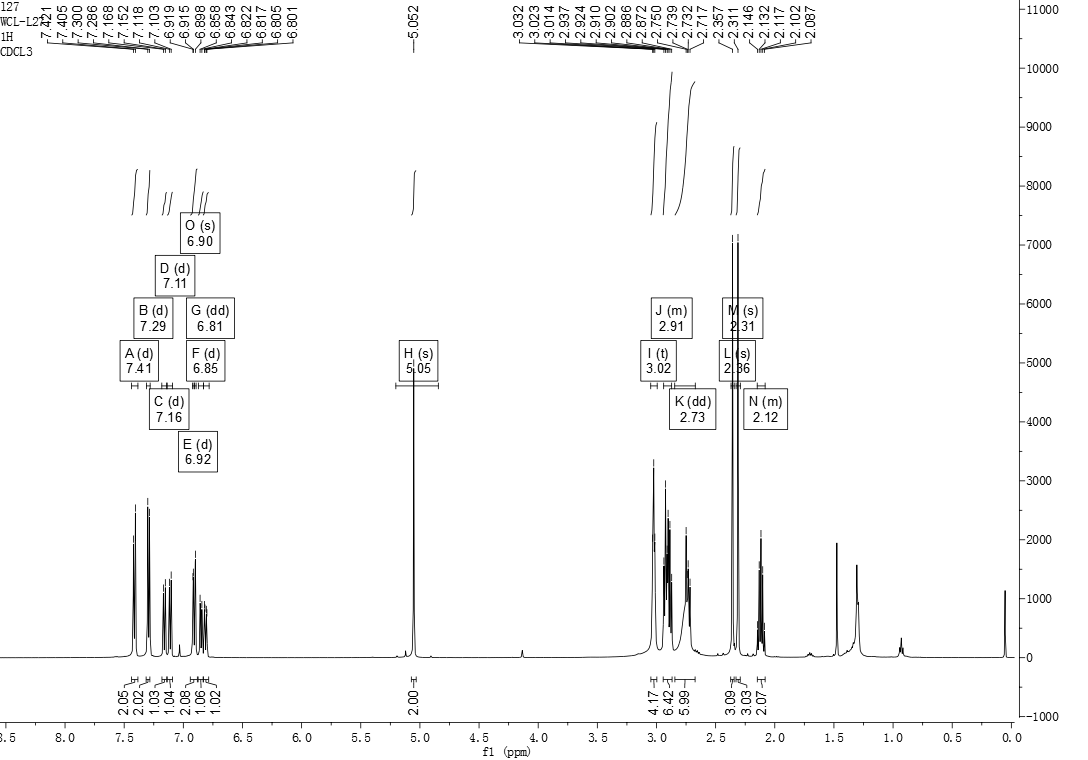


^1^H NMR spectrum of compound **11**


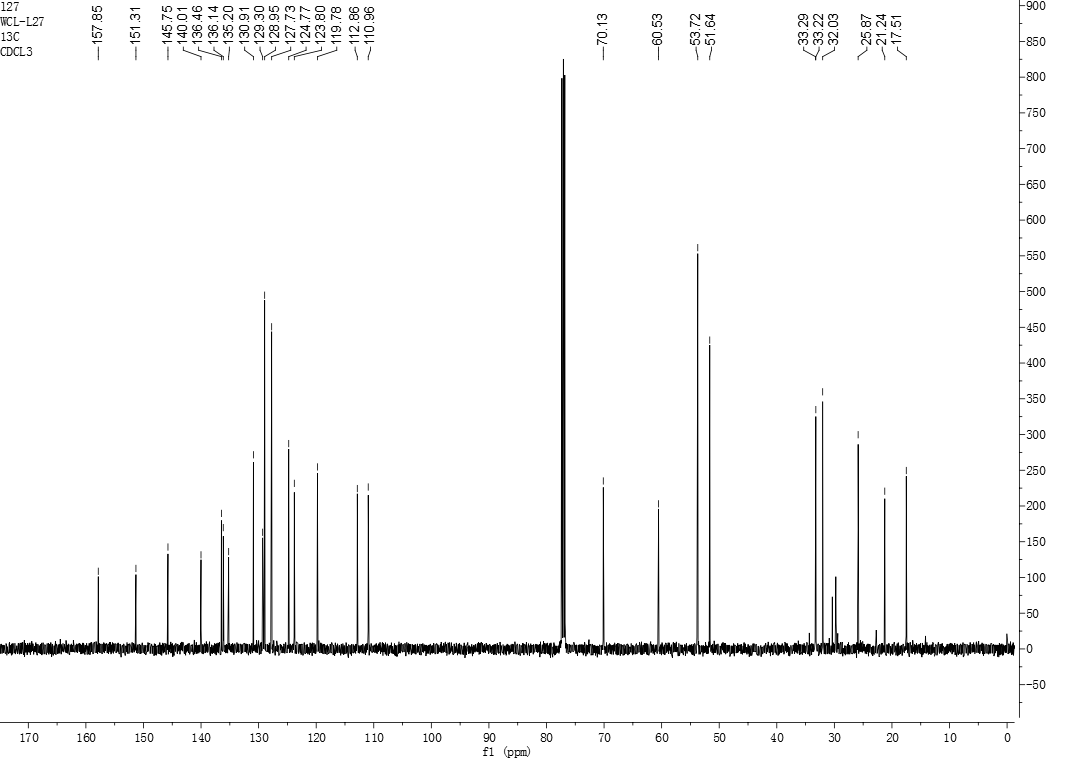


^13^C NMR spectrum of compound **11**

HRMS of compound **11**

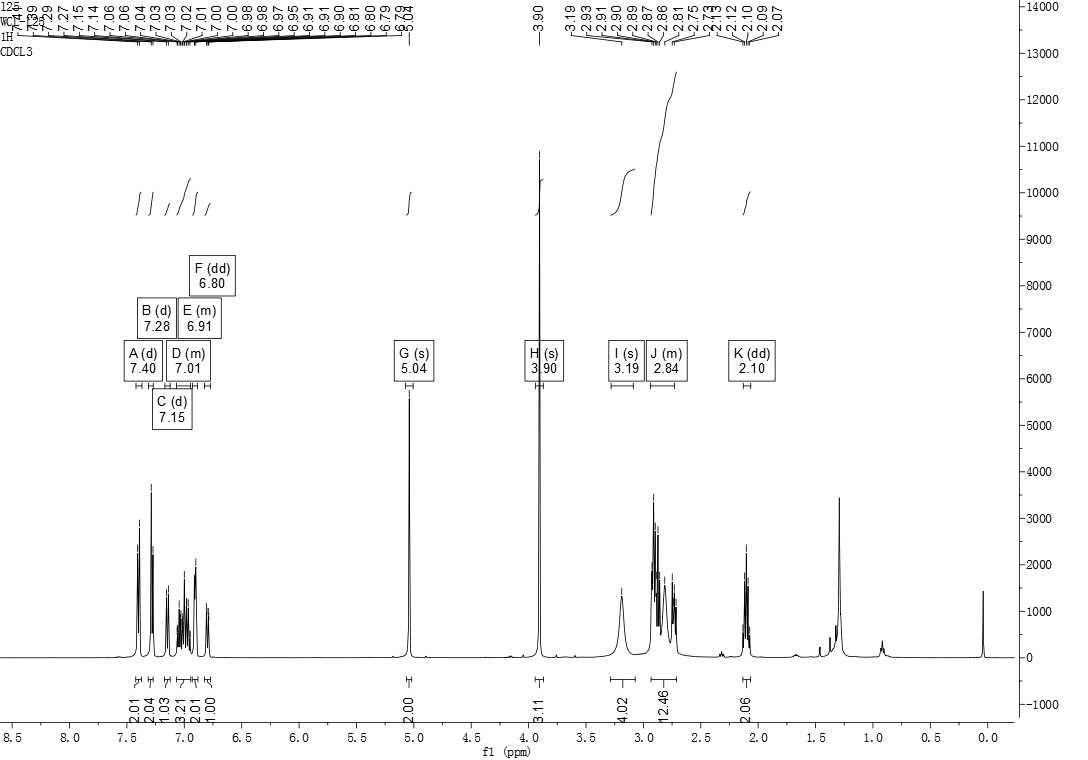


^1^H NMR spectrum of compound **12**


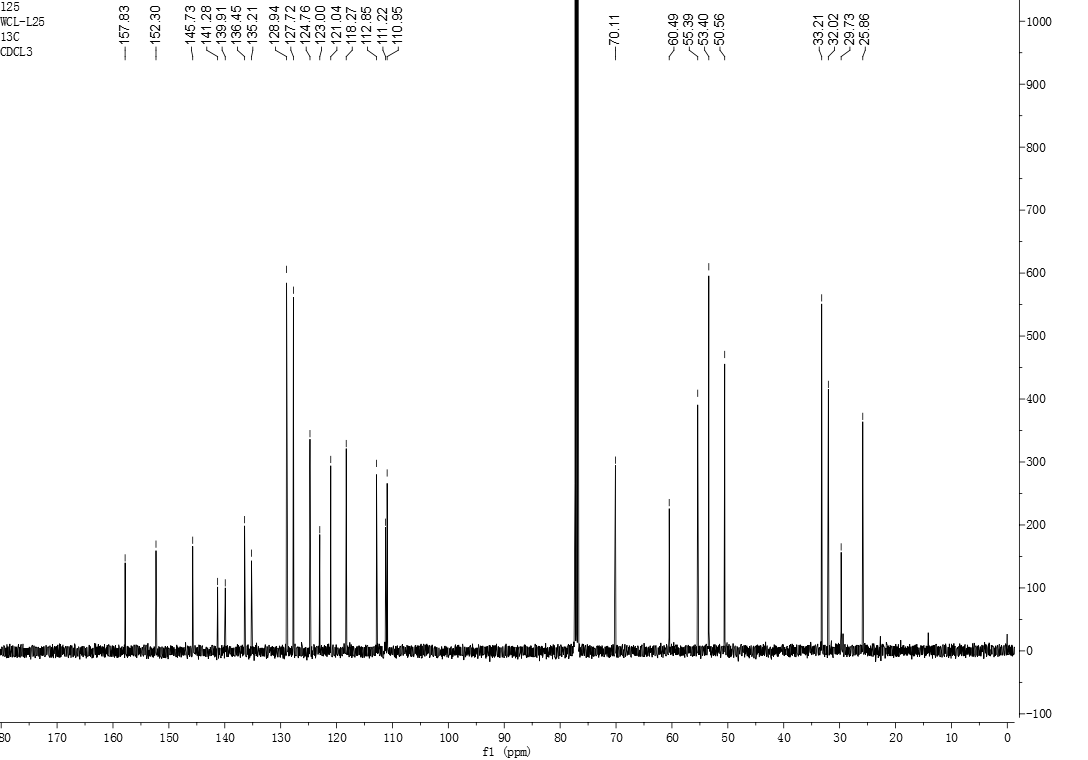


^13^C NMR spectrum of compound **12**

HRMS of compound **12**

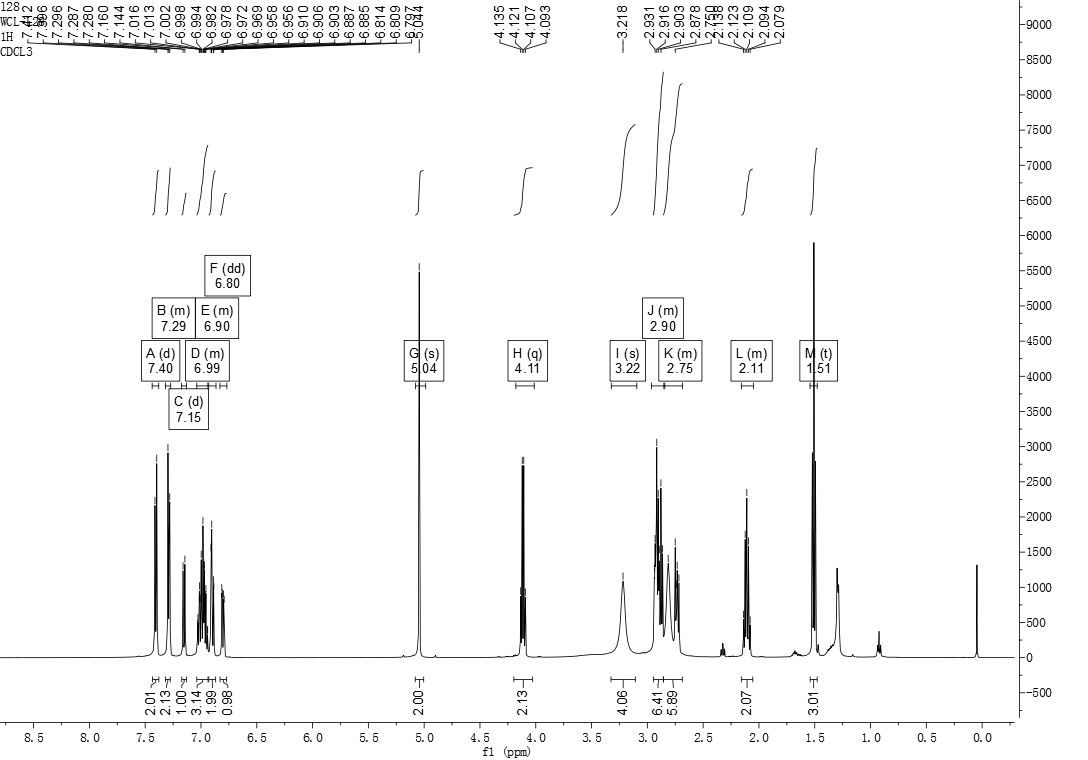


^1^H NMR spectrum of compound **13**


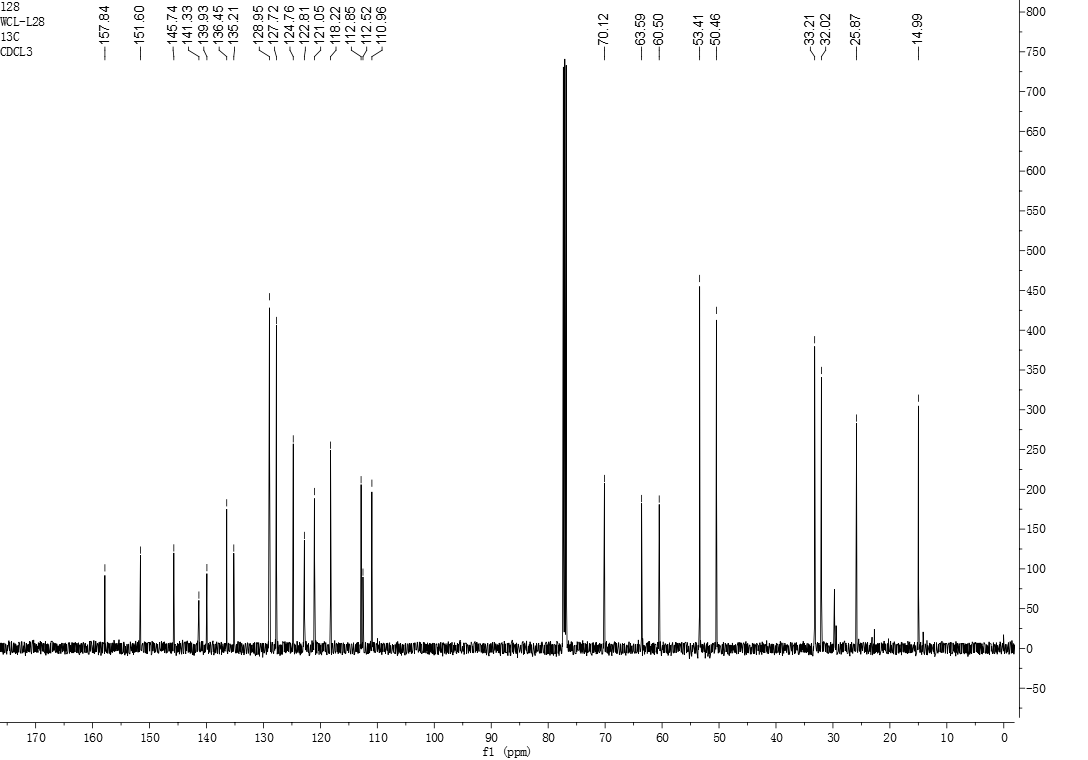


^13^C NMR spectrum of compound **13**

HRMS of compound **13**

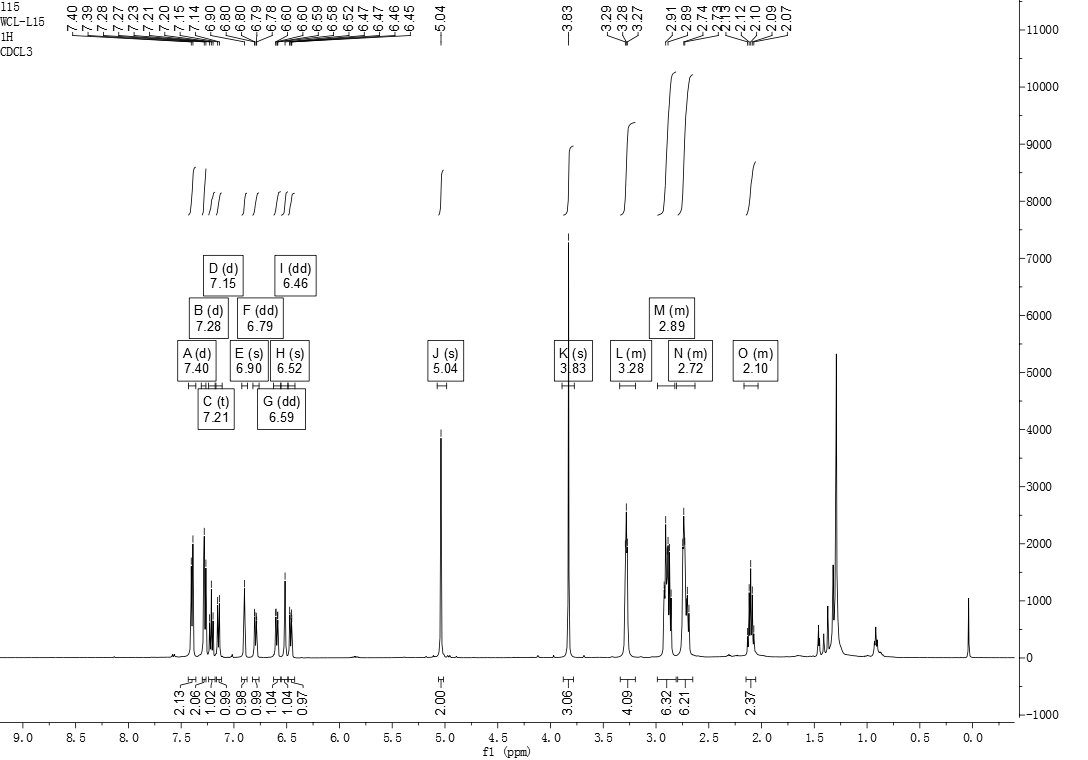


^1^H NMR spectrum of compound **14**


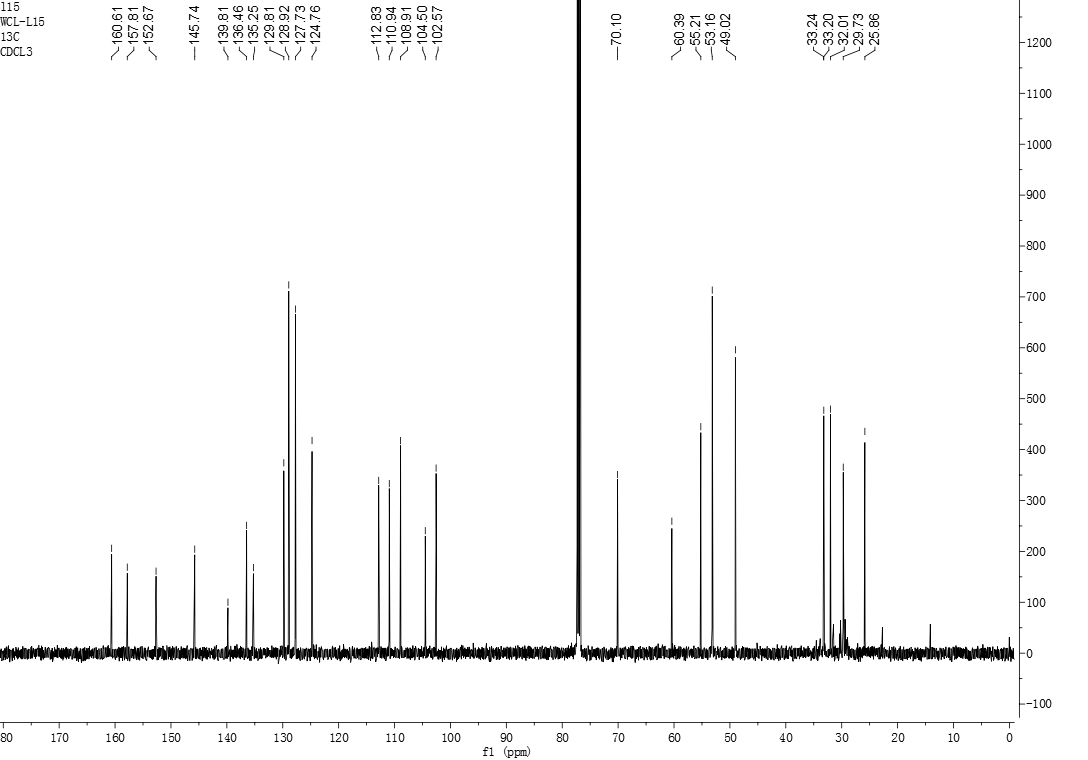


^13^C NMR spectrum of compound **14**

HRMS of compound **14**

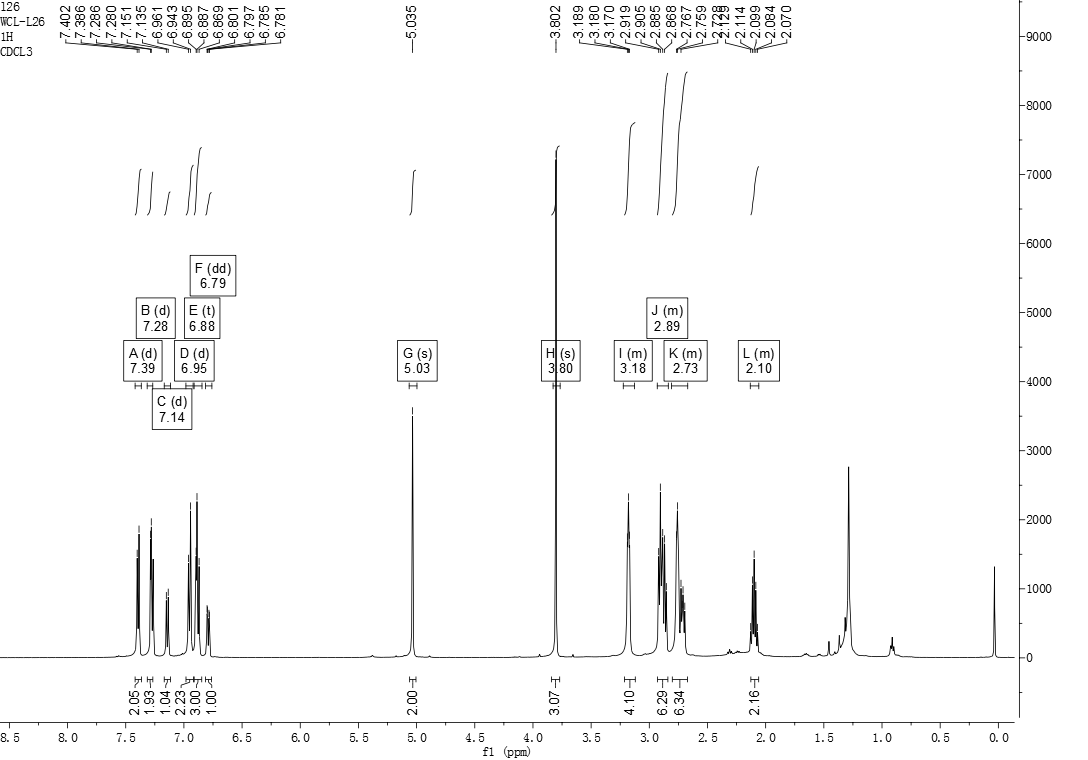


^1^H NMR spectrum of compound **15**


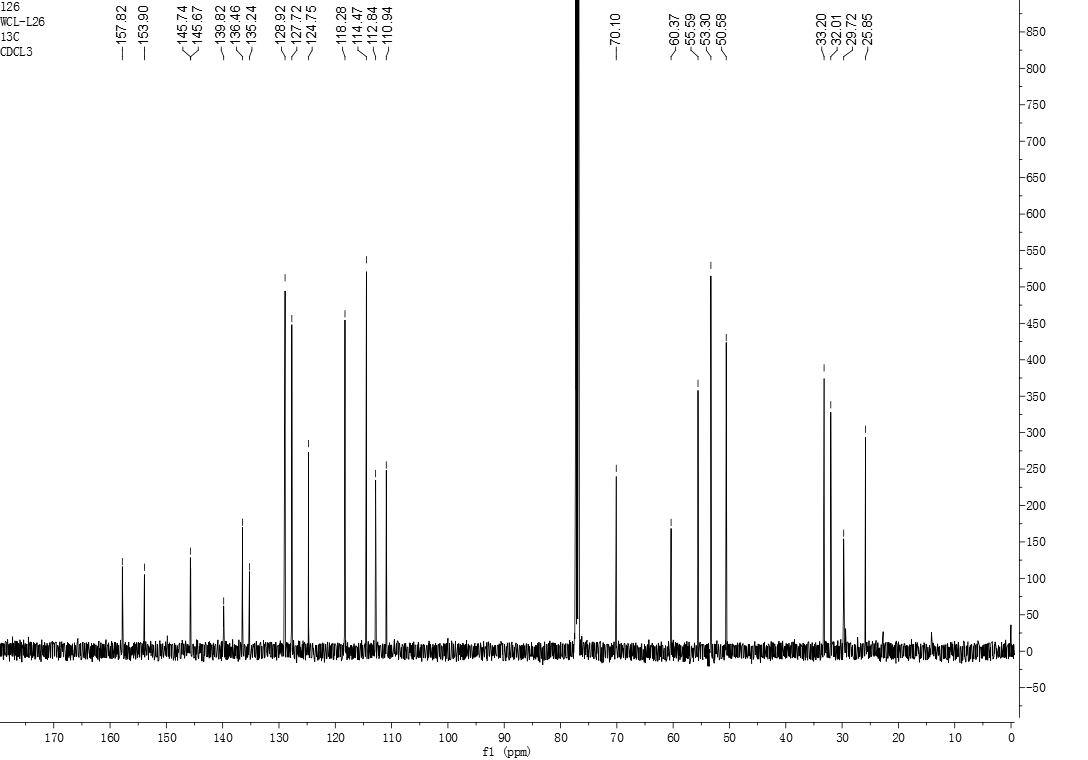


^13^C NMR spectrum of compound **15**

HRMS of compound **15**

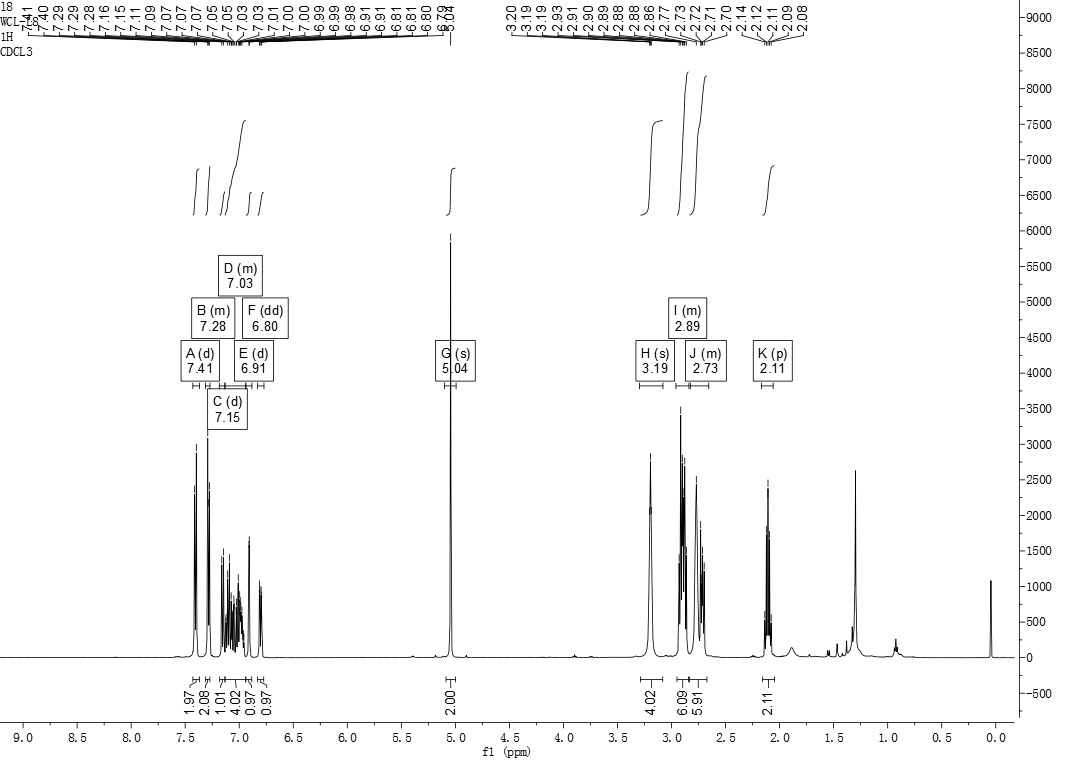


^1^H NMR spectrum of compound **16**


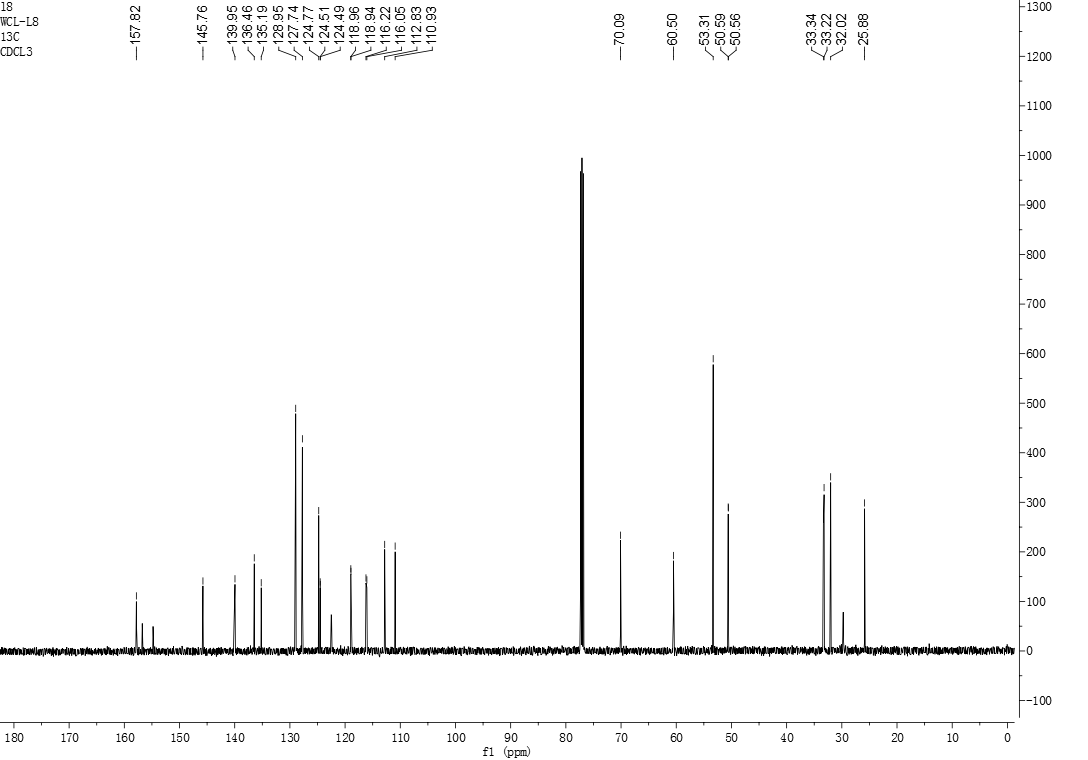


^13^C NMR spectrum of compound **16**

HRMS of compound **16**

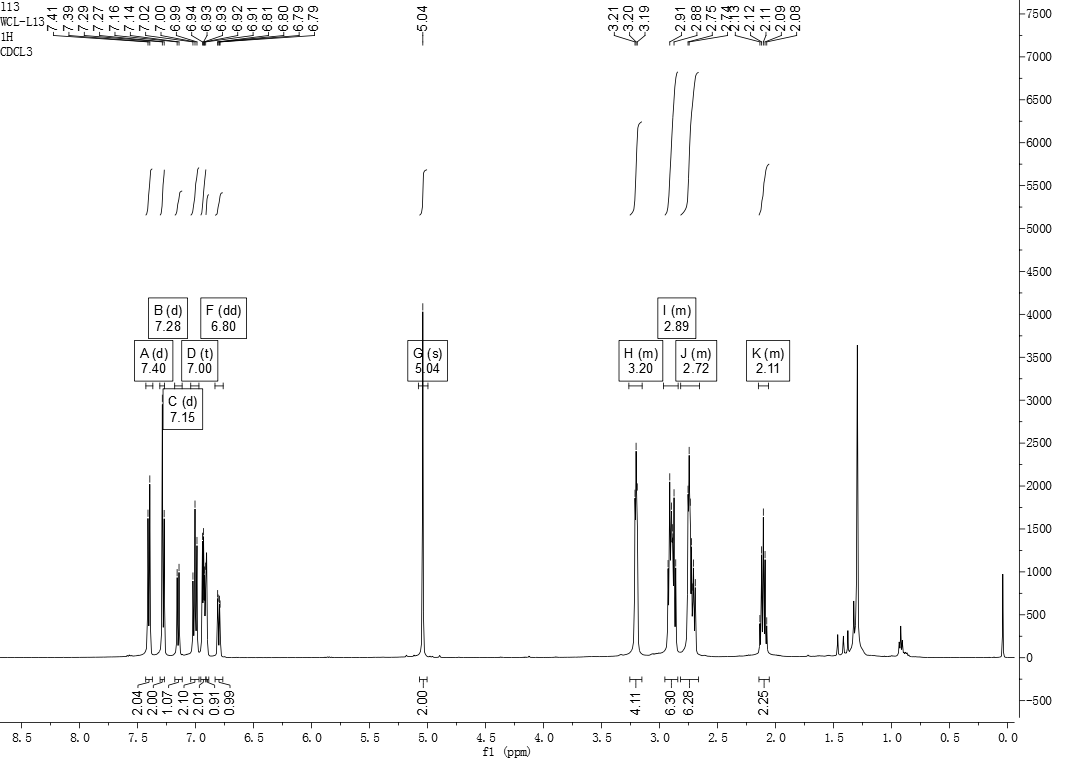


^1^H NMR spectrum of compound **17**


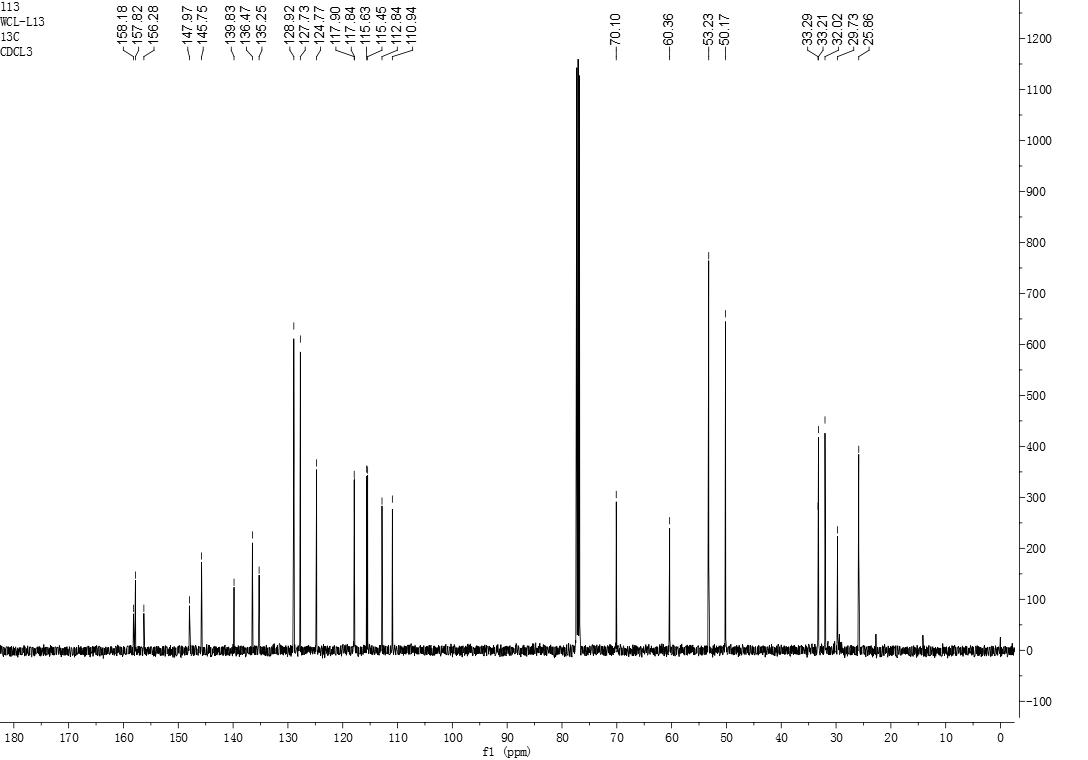


^13^C NMR spectrum of compound **17**

HRMS of compound **17**

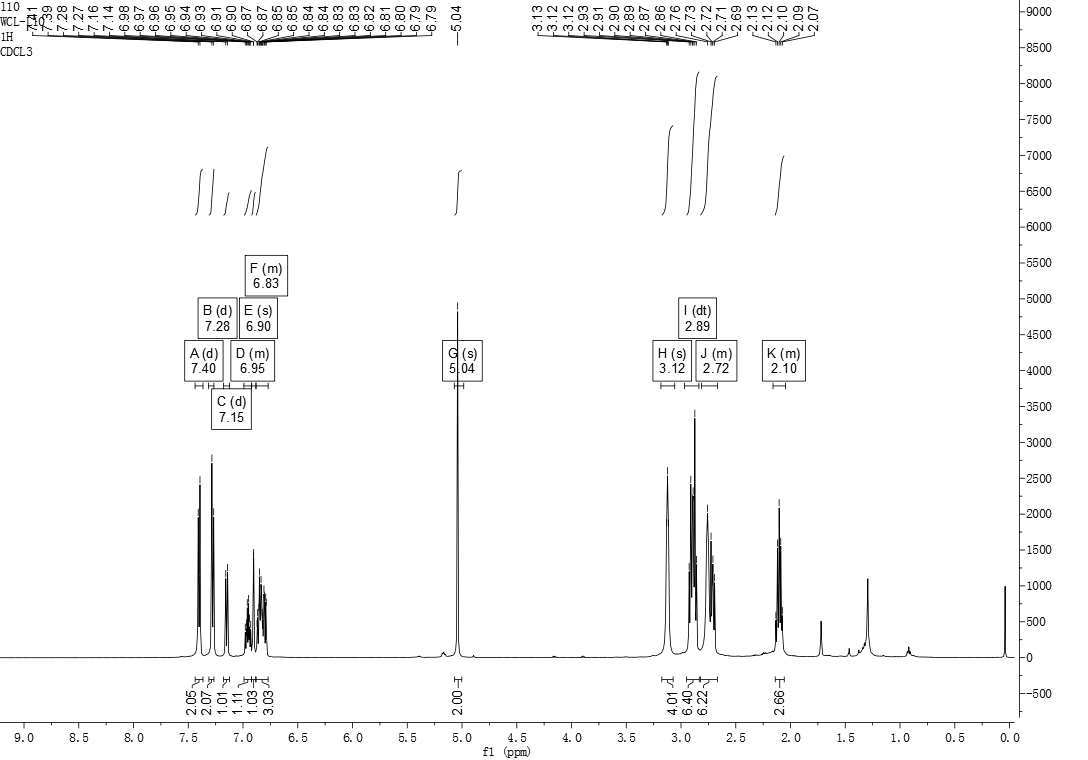


^1^H NMR spectrum of compound **18**


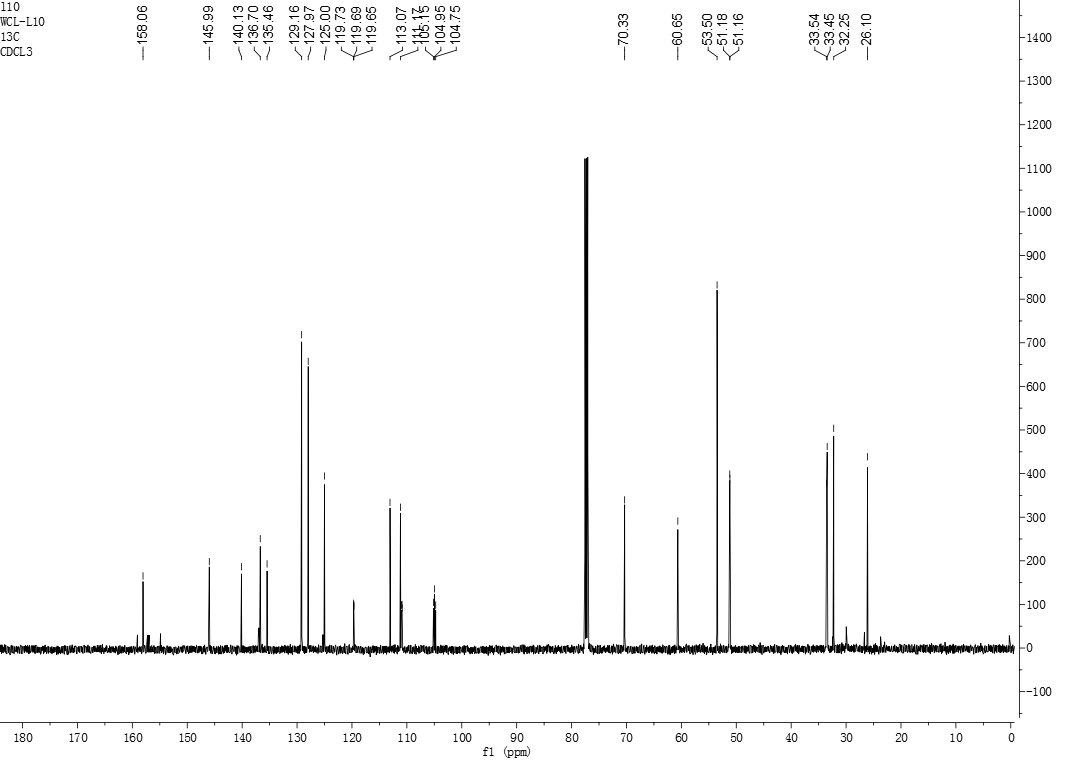


^13^C NMR spectrum of compound **18**

HRMS of compound **18**

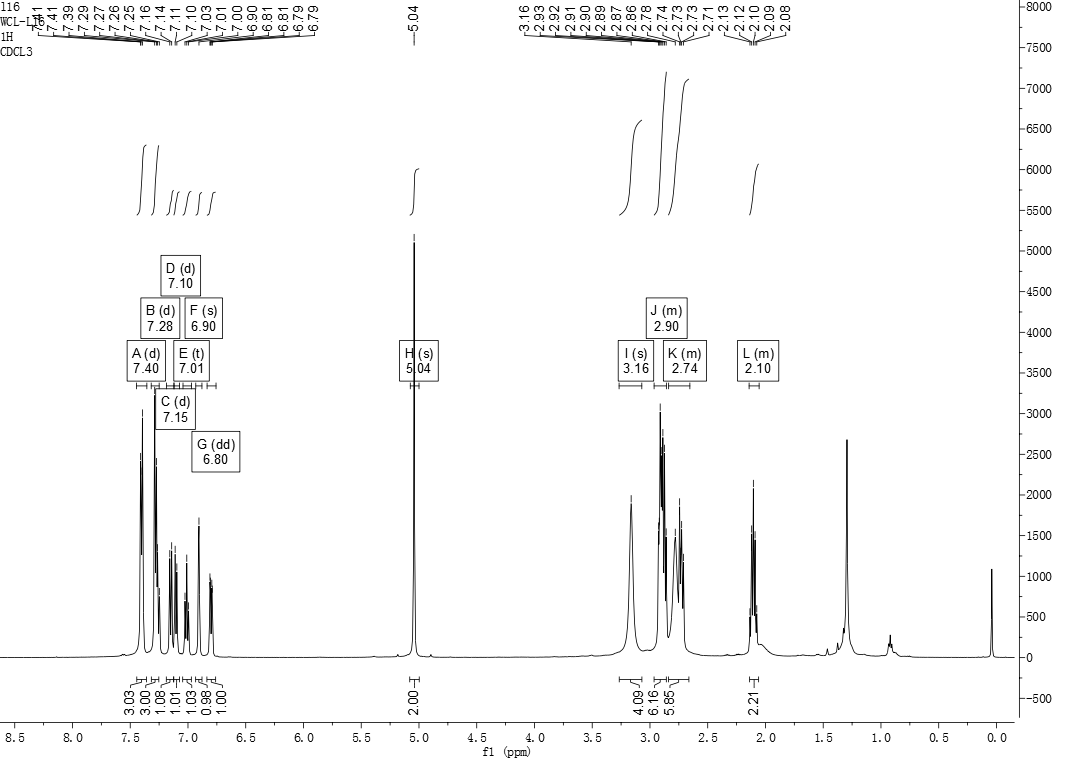


^1^H NMR spectrum of compound **19**


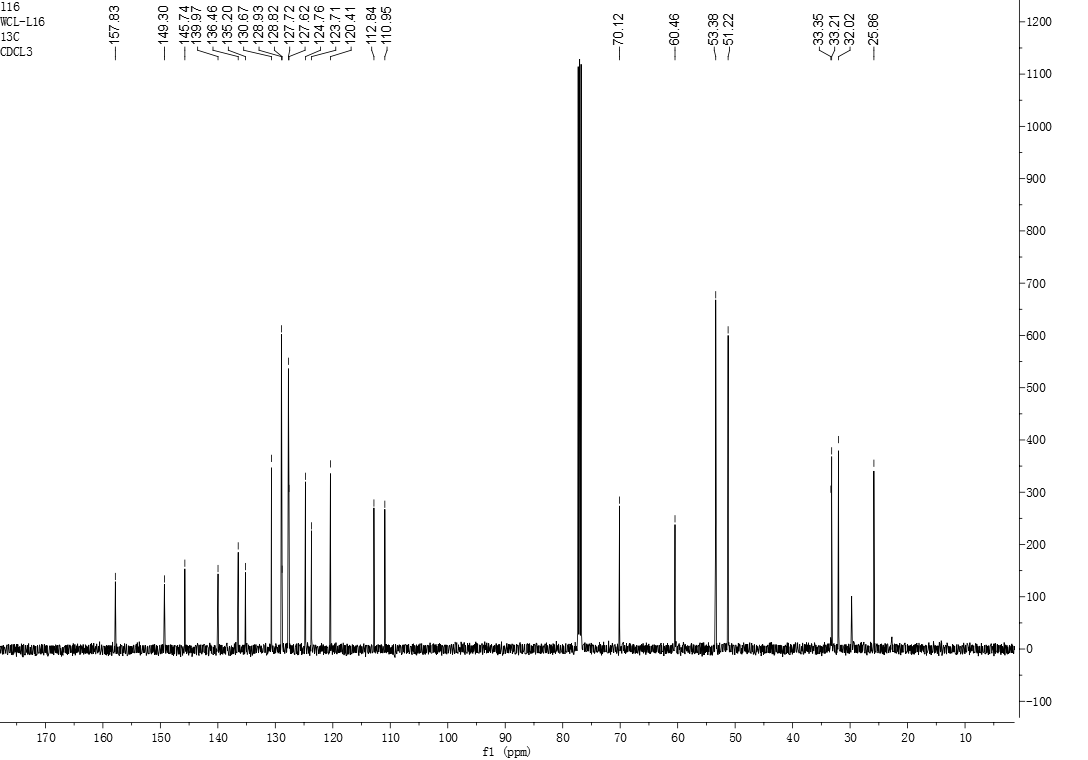


^13^C NMR spectrum of compound **19**

HRMS of compound **19**

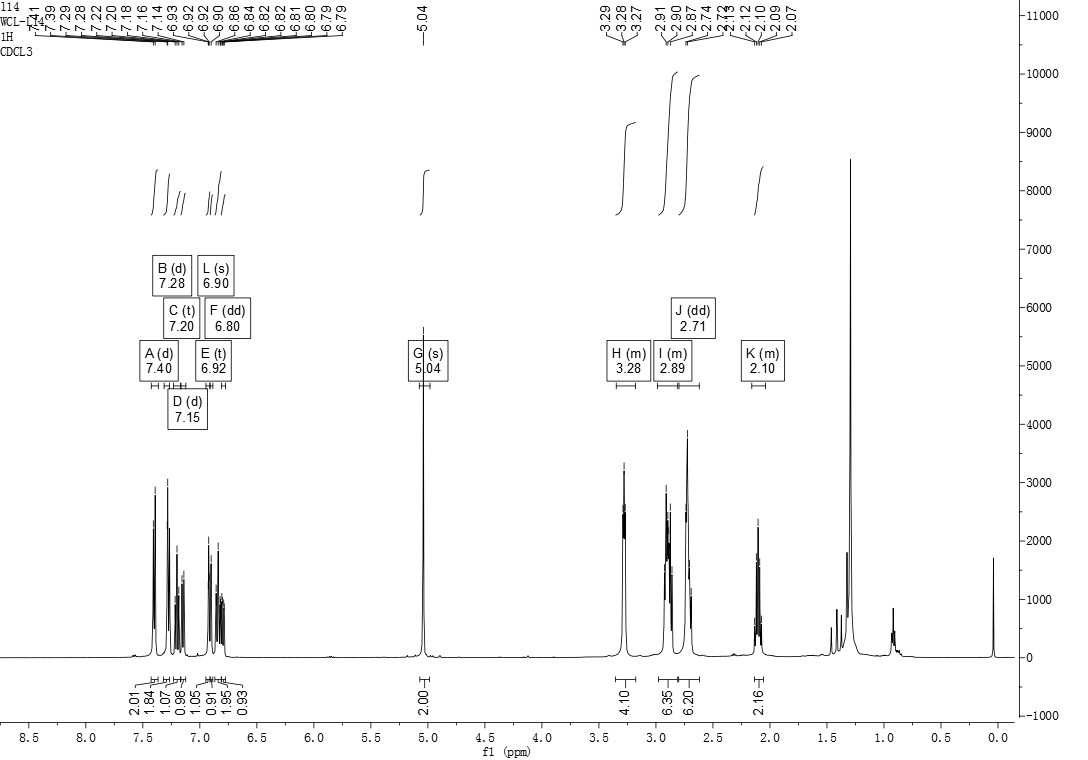


^1^H NMR spectrum of compound **20**


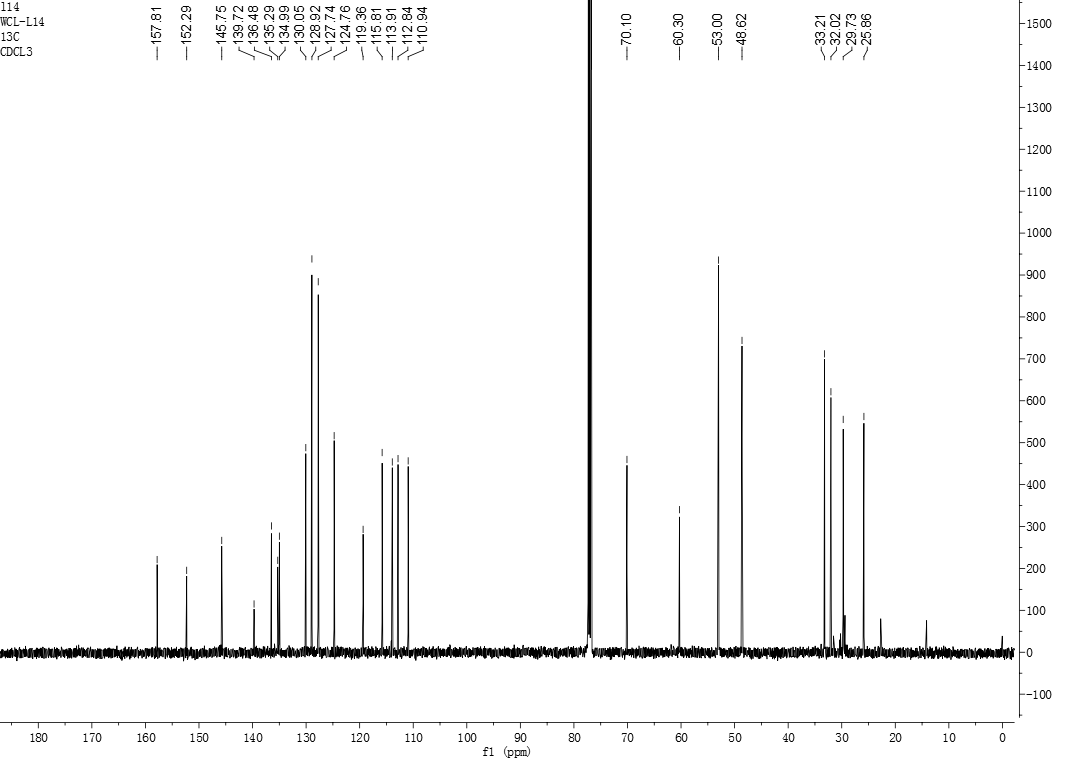


^13^C NMR spectrum of compound **20**

HRMS of compound **20**

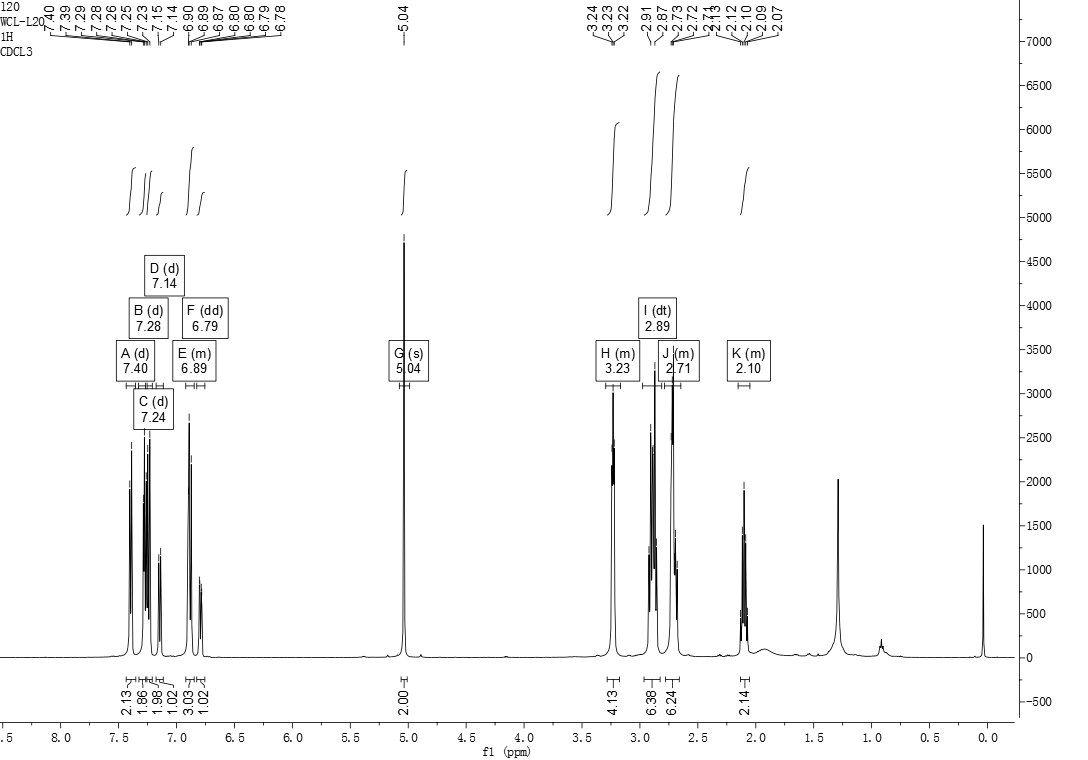


^1^H NMR spectrum of compound **21**


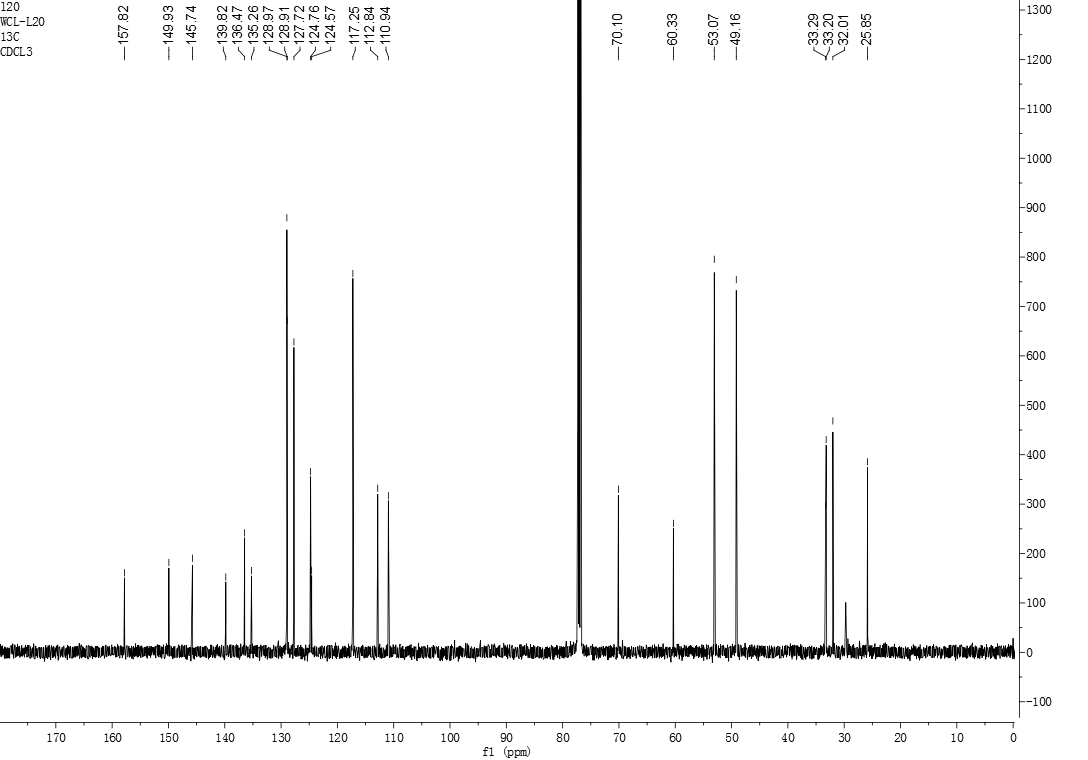


^13^C NMR spectrum of compound **21**

HRMS of compound **21**

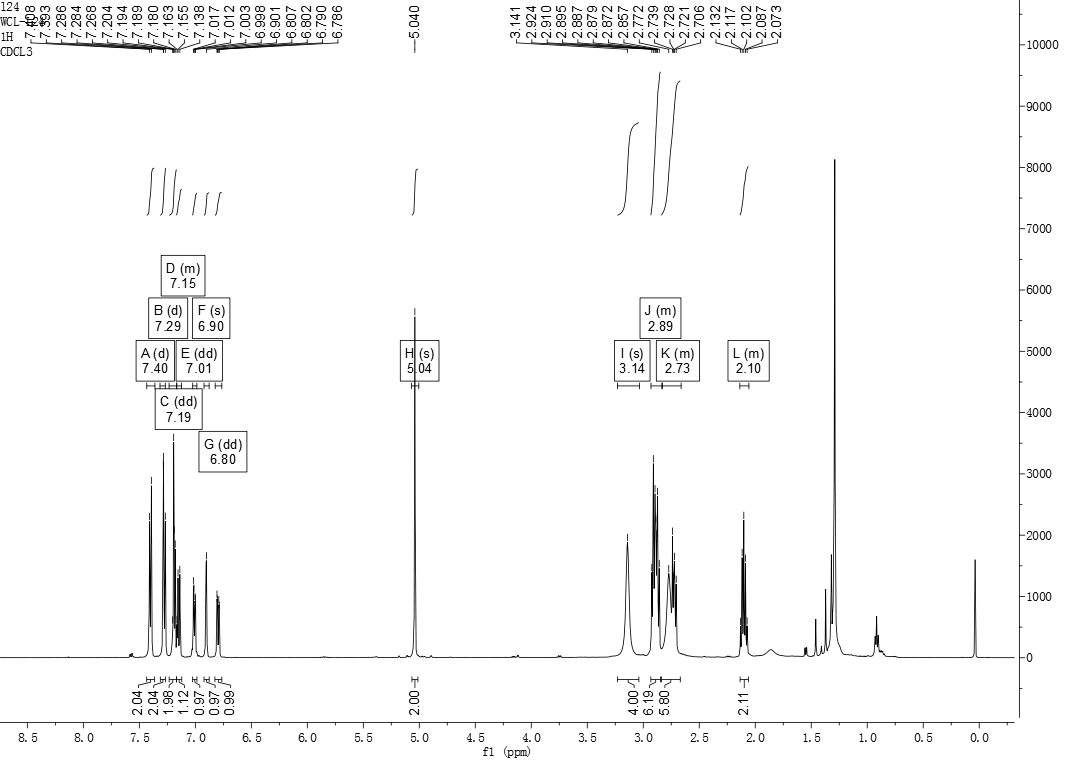


^1^H NMR spectrum of compound **22**


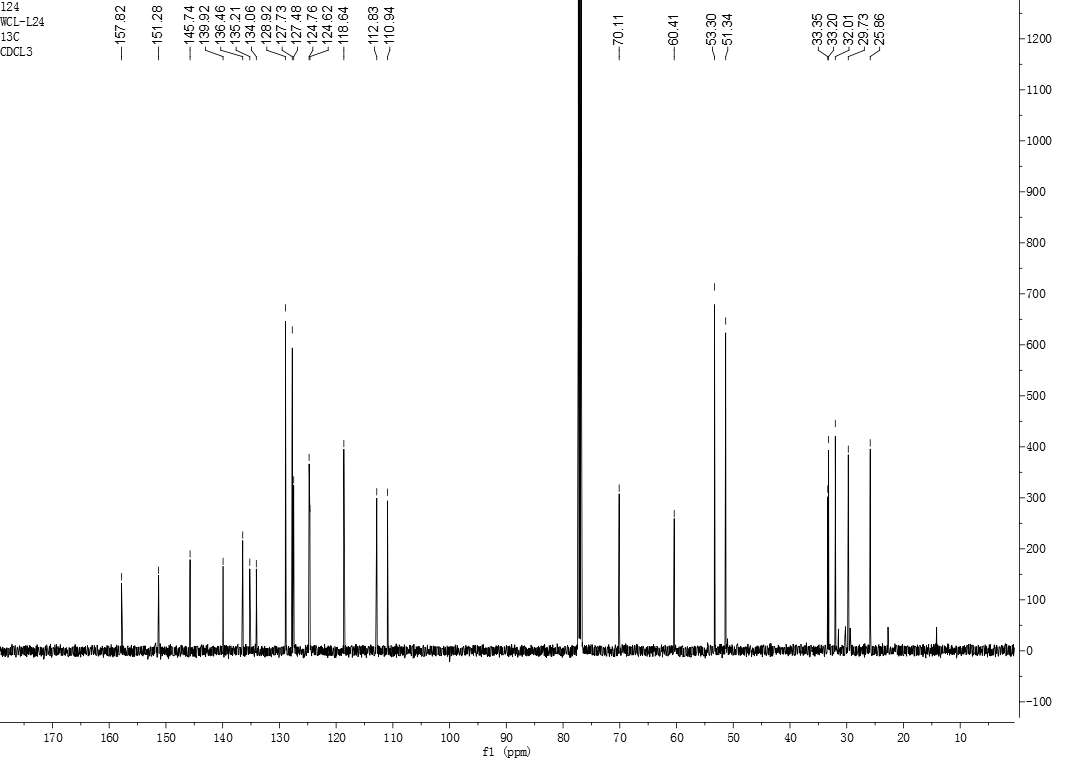


^13^C NMR spectrum of compound **22**

HRMS of compound **22**

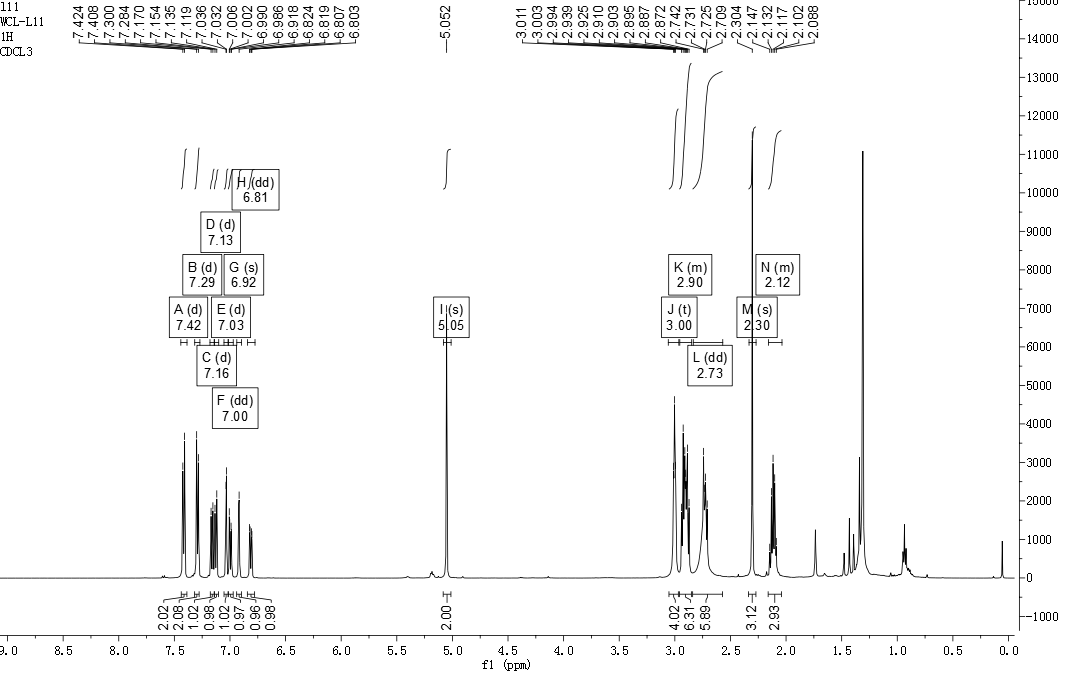


^1^H NMR spectrum of compound **23**


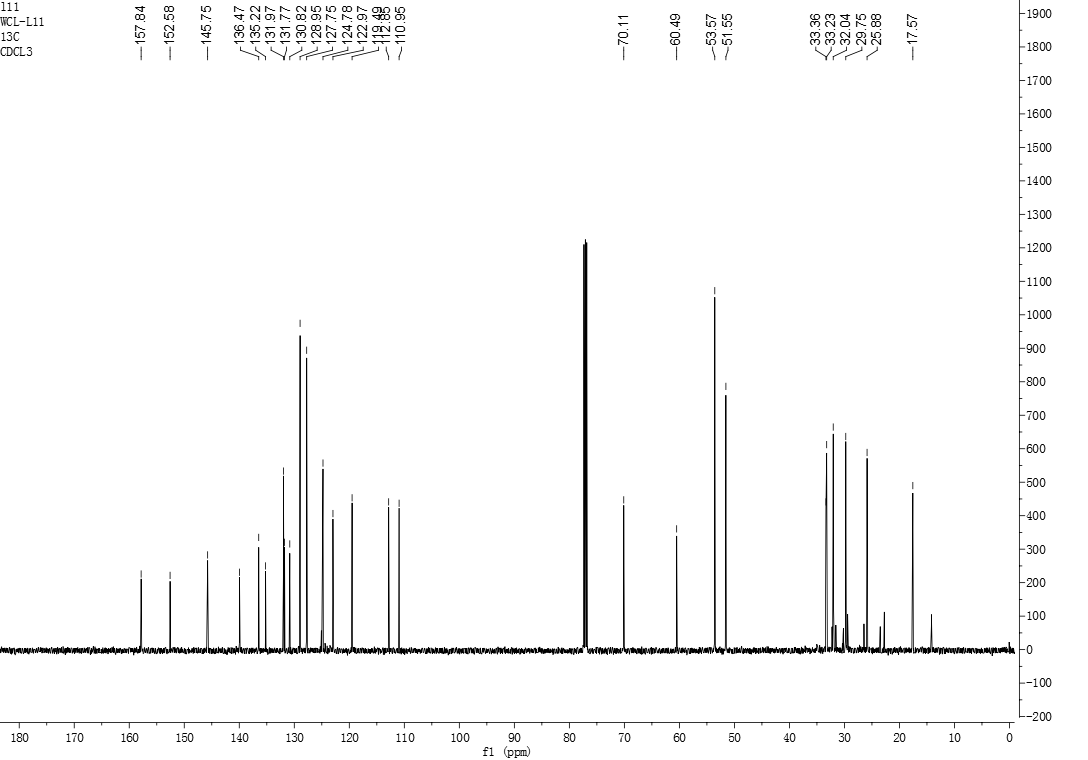


^13^C NMR spectrum of compound **23**

HRMS of compound **23**

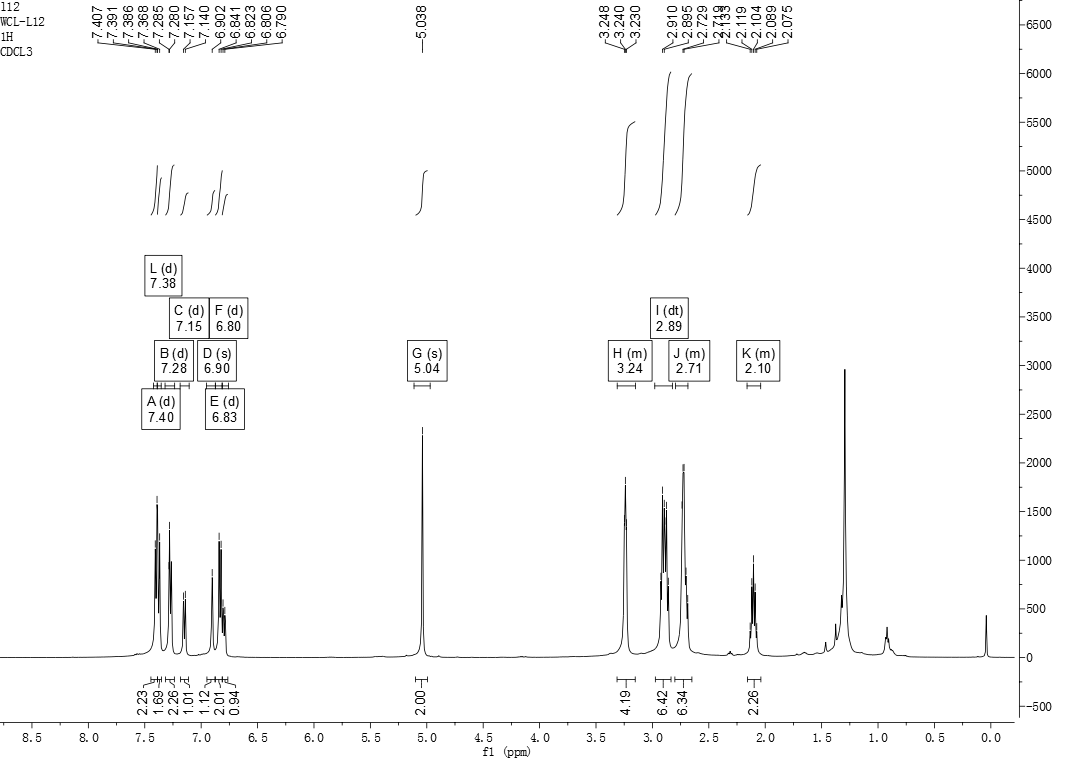


^1^H NMR spectrum of compound **24**


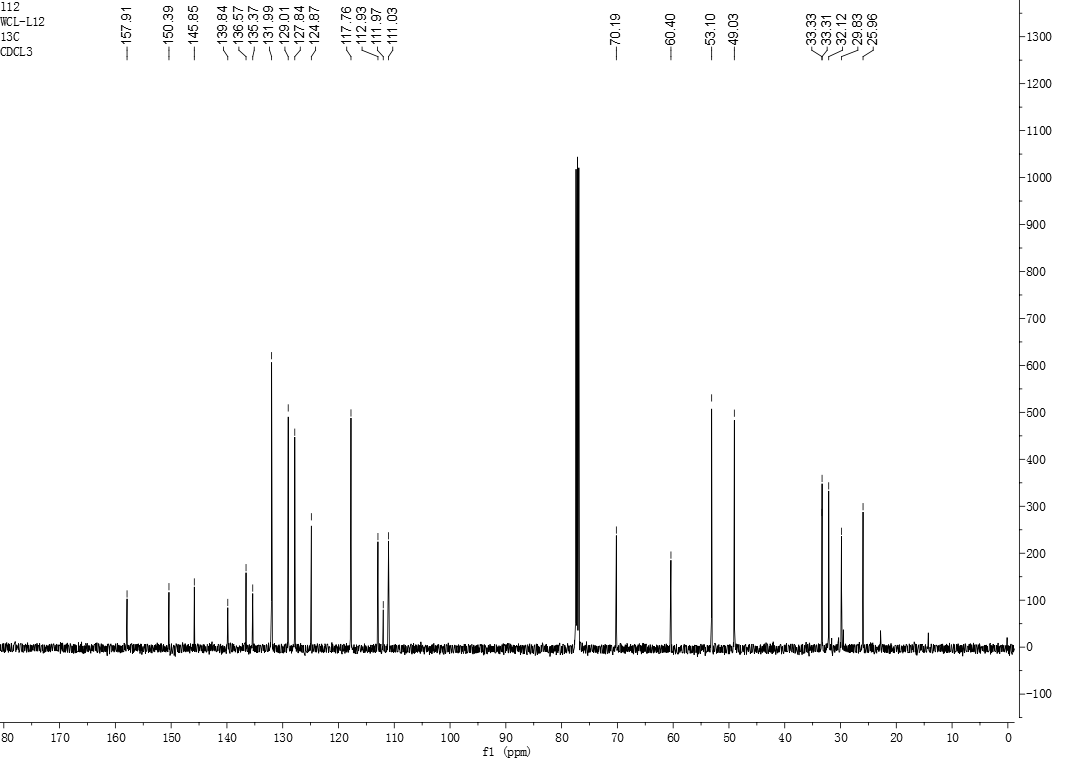


^13^C NMR spectrum of compound **24**

HRMS of compound **24**

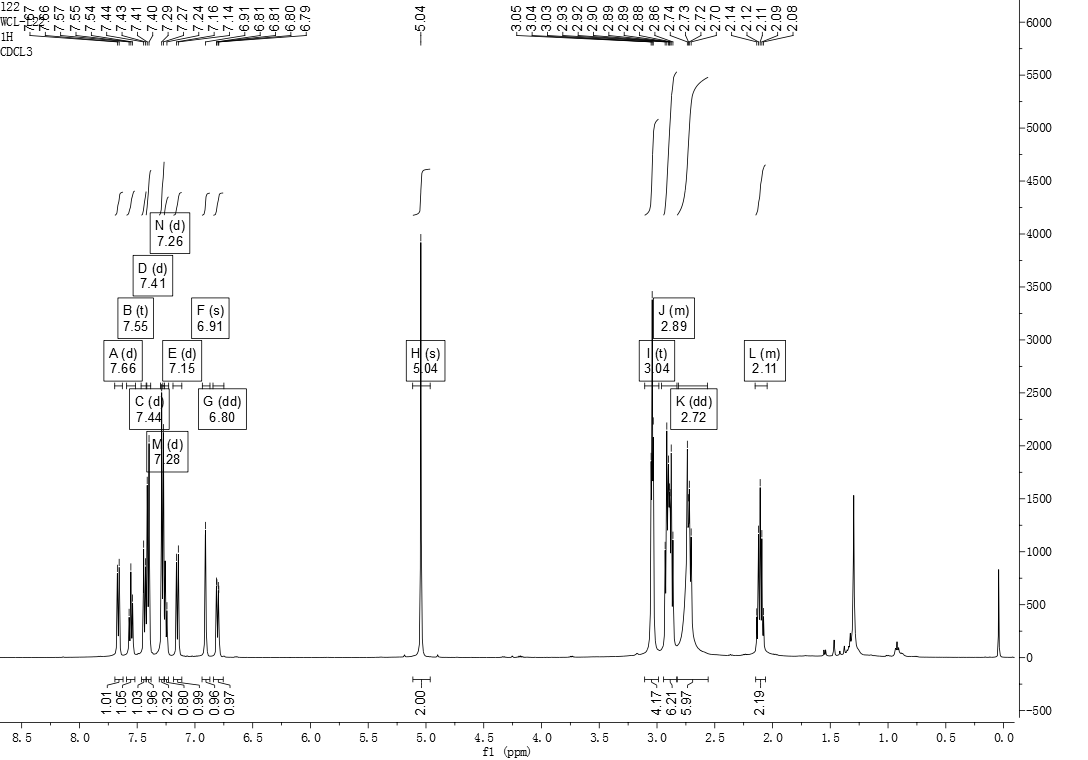


^1^H NMR spectrum of compound **25**


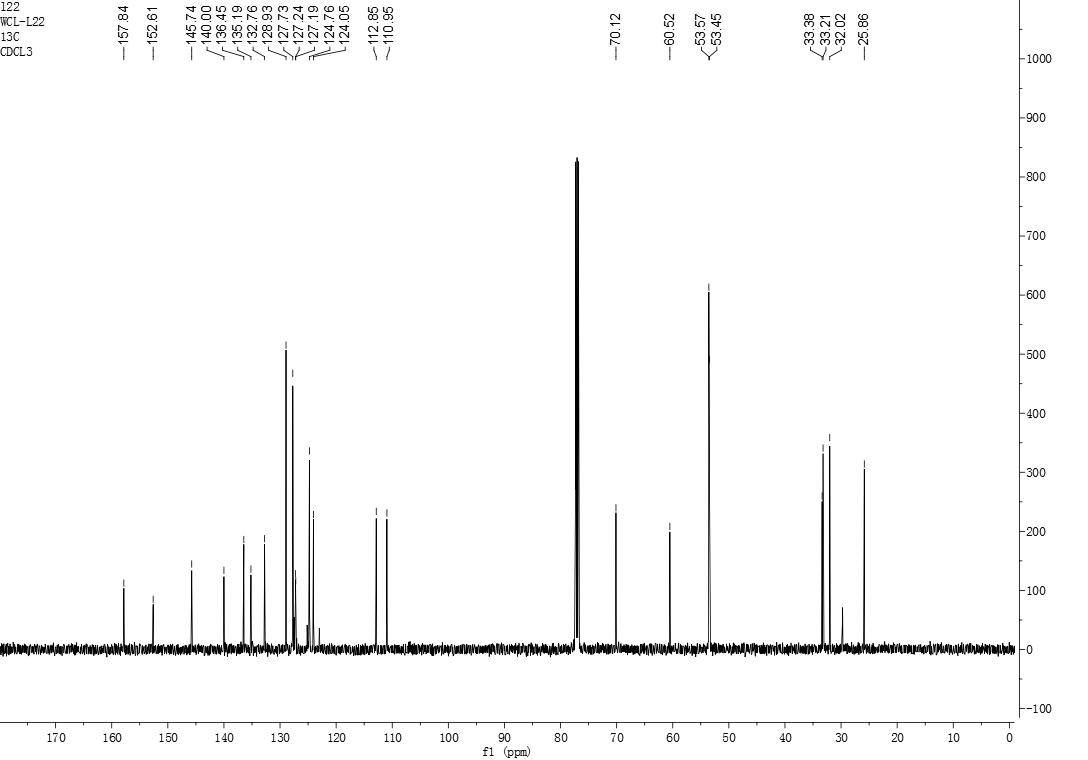


^13^C NMR spectrum of compound **25**

HRMS of compound **25**

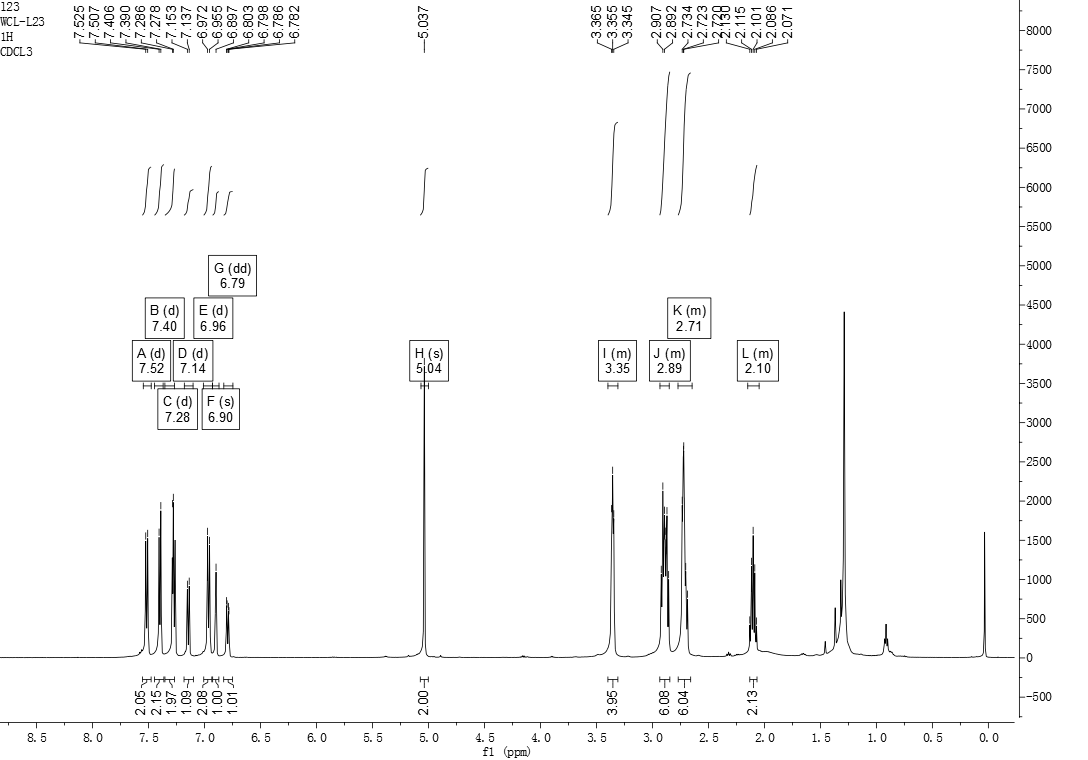


^1^H NMR spectrum of compound **26**


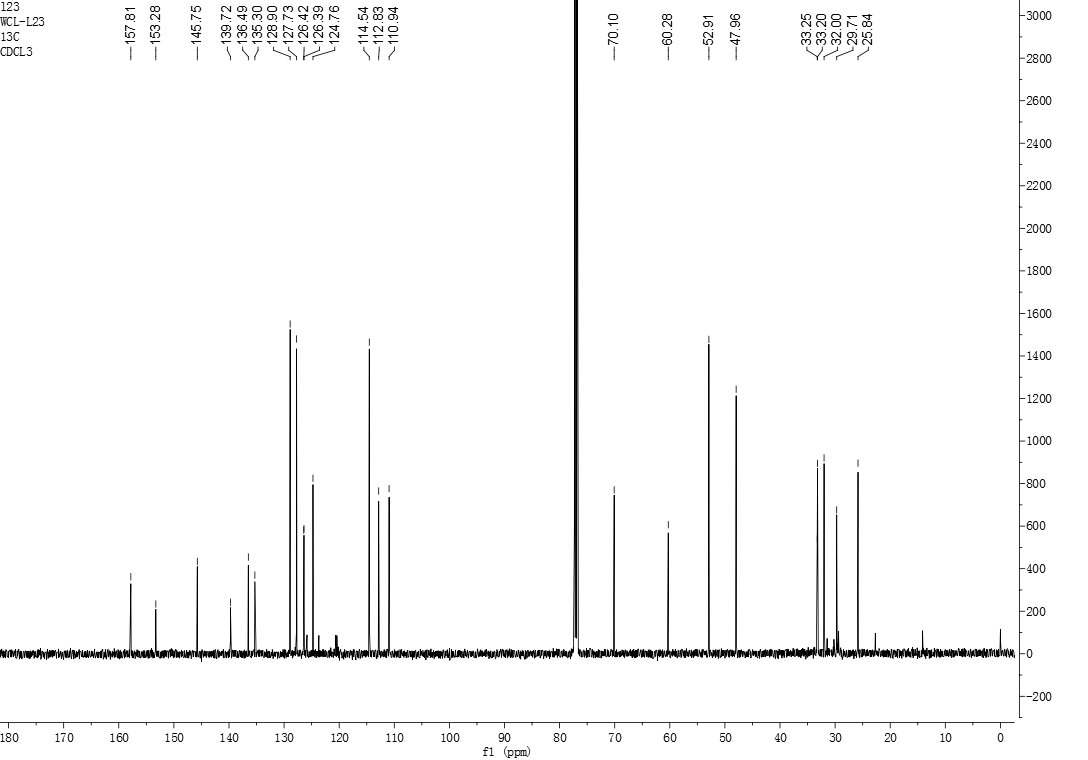


^13^C NMR spectrum of compound **26**

HRMS of compound **26**

1. [↑](#footnote-ref-0)
2. [↑](#footnote-ref-1)
3. [↑](#footnote-ref-2)
