# Supplementary figures and images for: Synthesis, bioactivity, and molecular docking of novel arylpiperazine derivatives as potential AR antagonists
Source: Front Chem. 2022 Aug 15;10:947065. doi: 10.3389/fchem.2022.947065 (PMC9420858; doi:10.3389/fchem.2022.947065)

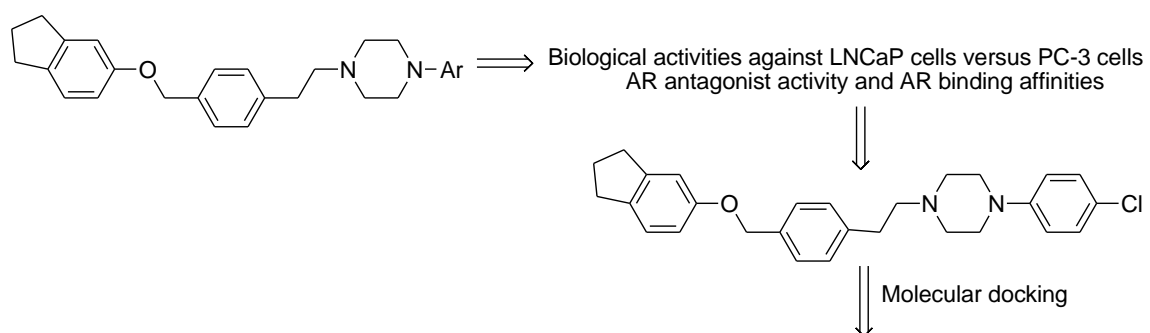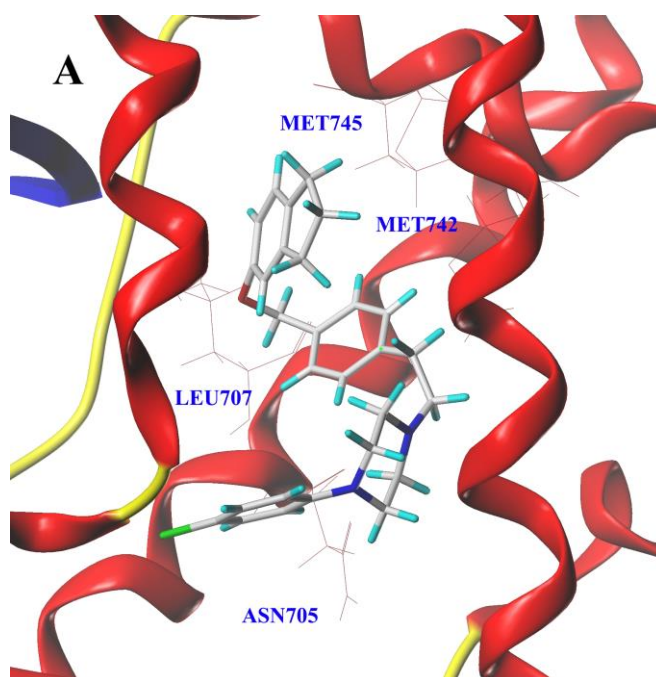

Supplement: Supplementary file 2 [file Image1.pdf]
